# Natural Killer Cell Activation by Ubiquitin-specific Protease 6 Mediates Tumor Suppression in Ewing Sarcoma

**DOI:** 10.1158/2767-9764.CRC-22-0505

**Published:** 2023-08-22

**Authors:** Kanika Jain, Ian C. Henrich, Laura Quick, Robert Young, Shreya Mondal, Andre M. Oliveira, Gerd A. Blobel, Margaret M. Chou

**Affiliations:** 1 Department of Pathology and Laboratory Medicine, Children's Hospital of Philadelphia, Philadelphia, Pennsylvania.; 2 Perelman School of Medicine at University of Pennsylvania, Philadelphia, Pennsylvania.; 3 Department of Laboratory Medicine and Pathology, Mayo Clinic, Rochester, Minnesota.; 4 Department of Pediatric Hematology, Children's Hospital of Philadelphia, Philadelphia, Pennsylvania.

## Abstract

Ewing sarcoma is a rare and deadly pediatric bone cancer for which survival rates and treatment options have stagnated for decades. Ewing sarcoma has not benefited from immunotherapy due to poor understanding of how its immune landscape is regulated. We recently reported that ubiquitin-specific protease 6 (USP6) functions as a tumor suppressor in Ewing sarcoma, and identified it as the first cell-intrinsic factor to modulate the Ewing sarcoma immune tumor microenvironment (TME). USP6 induces intratumoral infiltration and activation of multiple innate immune lineages in xenografted nude mice. Here we report that natural killer (NK) cells are essential for its tumor-inhibitory functions, as NK cell depletion reverses USP6-mediated suppression of Ewing sarcoma xenograft growth. USP6 expression in Ewing sarcoma cells directly stimulates NK cell activation and degranulation *in vitro*, and functions by increasing surface levels of multiple NK cell-activating ligands. USP6 also induces surface upregulation of the receptor for the apoptosis-inducing ligand tumor necrosis factor-related apoptosis-inducing ligand (TRAIL), providing an additional route for enhanced sensitivity to NK cell killing. Furthermore, USP6-expressing Ewing sarcoma and NK cells participate in a paracrine immunostimulatory feedforward loop, wherein IFNγ secreted by activated NK cells feeds back on USP6/Ewing sarcoma cells to induce synergistic expression of chemokines CXCL9 and CXCL10. Remarkably, expression of USP6 in subcutaneous Ewing sarcoma xenografts induces systemic activation and maturation of NK cells, and induces an abscopal response in which growth of distal tumors is inhibited, coincident with increased infiltration and activation of NK cells. This work reveals how USP6 reprograms the Ewing sarcoma TME to enhance antitumor immunity, and may be exploited for future therapeutic benefit.

**Significance:**

This study provides novel insights into the immunomodulatory functions of USP6, the only cancer cell–intrinsic factor demonstrated to regulate the immune TME in Ewing sarcoma. We demonstrate that USP6-mediated suppression of Ewing sarcoma tumorigenesis is dependent on NK cells. USP6 directly activates NK cell cytolytic function, inducing both intratumoral and systemic activation of NK cells in an Ewing sarcoma xenograft model.

## Introduction

Ewing sarcoma (ES) is a rare pediatric malignancy, representing the second most common bone cancer, with approximately 200 cases per year in the United States (1–3). Ewing sarcoma predominantly afflicts children between 11 and 20 years of age. While the 5-year survival rate for patients with localized tumors is 70%, the rate plummets to less than 25% upon recurrence or metastases, despite multimodal therapy that includes chemotherapy, radiation, and surgery. Metastatic lesions can arise in lung, bone, or bone marrow, with pulmonary tumors being the leading cause of mortality. The key etiologic driver in Ewing sarcoma are chromosomal translocations between *EWS* and *Ets* family transcription factors, most commonly t(11;22)(q24;q12), which generates the *EWS-FLI1* fusion (1–3). EWS-FLI1 can function as a pioneer transcription factor by binding to genomic elements enriched in polymorphic GGAA microsatellites, inducing chromatin reorganization and formation of *de novo* enhancers and superenhancers (4, 5). Numerous groups have attempted to target this chimera or critical downstream effectors therapeutically; however, none have been clinically successful, underscoring the need for alternate strategies (6).

While immunotherapy has had remarkable impact on patient survival in numerous malignancies, it has not been successful in Ewing sarcoma (7–10). Ewing sarcoma possesses one of the lowest mutational burdens among all cancers, and expresses a paucity of tumor-specific antigens. Its immune landscape comprises predominantly protumorigenic M2 macrophages and typically low levels of CD8^+^ T lymphocytes. Furthermore, a key mechanism of immune escape in Ewing sarcoma arises from impaired expression of MHC class I (MHCI)/HLA class I, with a significant fraction of primary and metastatic lesions expressing little to no class I and II MHC (11, 12). Accordingly, immune checkpoint inhibitor (ICI) therapy has not been successfully utilized in Ewing sarcoma (7–10). Identifying means to stimulate intratumoral infiltration of CD8^+^ T cells and to increase surface expression of MHCI/II could promote ICI responsiveness in patients with Ewing sarcoma. However, it is also essential to develop immunotherapeutic strategies that target alternative cytolytic immune lineages that can kill in a MHCI nonrestricted manner, such as natural killer (NK) cells, the chief cytolytic innate immune lineage (13, 14). Numerous studies point to a central role for NK cells in combatting progression and metastasis of both sarcomas and many other cancers (13–18). A high frequency of intratumoral activated NK cells has been associated with significantly improved overall survival in Ewing sarcoma (19). Furthermore, prior work has shown that NK cell–based adoptive cell therapy can suppress growth of primary tumors (PT) and lung metastases in mouse models of Ewing sarcoma (16–18). However, Ewing sarcoma–intrinsic factors that regulate infiltration and activation of NK cells are completely unknown, and elucidation of these mechanisms could be highly informative for development of novel therapeutics.

We recently reported an unexpected tumor-suppressive role for the deubiquitinating enzyme ubiquitin-specific protease 6 (USP6) in Ewing sarcoma (20). High USP6 expression in patients with Ewing sarcoma was associated with significantly improved overall survival, as well as all molecular hallmarks of a “hot” tumor microenvironment (TME; ref. 20), defined as an IFN response gene signature, production of the chemokine CXCL10, and infiltration of CD8^+^ T lymphocytes (21). We discovered that USP6 was sufficient to directly induce the former two responses via deubiquitination and stabilization of Jak1, leading to phosphorylation and activation of STAT1 (20, 22, 23). USP6 also triggered surface stabilization of the type I and type II IFN receptors, IFNAR1 and IFNGR1, rendering Ewing sarcoma cells hyperresponsive to ectopic IFNs with resultant synergistic induction of IFN response genes, including *CXCL10* (20).

Strikingly, we observed that USP6 inhibited growth of Ewing sarcoma tumors when xenografted into nude but not NOD *scid* gamma (NSG) mice, implicating a role for the innate immune system in mediating the tumor-suppressive function of USP6 (20). In the current study, we demonstrate that NK cells are required for inhibition of Ewing sarcoma xenograft growth by USP6. Expression of USP6 in Ewing sarcoma cells directly induces NK cell activation by driving surface upregulation of NK-activating ligands. In addition, USP6 sensitizes Ewing sarcoma cells to killing by NK cells by increasing surface expression of the TRAIL receptor, DR5. Remarkably, USP6 expression in subcutaneous Ewing sarcoma xenografts induces systemic activation of NK cells in peripheral blood (PB) and also triggers an abscopal response against distal subcutaneous tumors. In sum, these results identify USP6 as the first Ewing sarcoma–intrinsic factor capable of directly activating antitumorigenic immunity, both intratumorally and systemically.

## Materials and Methods

### Cell Lines and Primary Cells

RD-ES were obtained from Dr. Frederic Barr (NCI, Bethesda, MD), and A673 were obtained from the ATCC. Their identity was confirmed upon initial receipt by short tandem repeat analysis using the University of Pennsylvania Genetics Core, and annually thereafter. RD-ES and A673 derivatives expressing USP6 in a doxycycline (dox)-inducible manner have been described previously (20, 23). RD-ES CRISPR lines were generated using Genscript pLentiCRISPRv2, targeting IFNAR1 (guide sequence GGCGTGTTTCCAGACTGTTT) and IFNGR1 (guide sequence ACATGAACCCTATCGTATAT). NK-92 were generously provided by Dr. Kerry Campbell (Fox Chase Cancer Center), and YAC1 were from ATCC. *Mycoplasma* screening of all cell lines was performed every 4–8 weeks using MycoAlert Detection Kit (Lonza). Experiments were performed on cell lines maintained for less than 10–20 passages after thawing. Primary human NK cells (obtained from the Human immunology Core, University of Pennsylvania, Philadelphia, PA) were isolated from PB mononuclear cells collected from healthy apheresis donors through negative selection using the RosetteSep Human NK Cell Enrichment Cocktail (Stem Cell Technologies, #15025). Primary mouse NK cells (lymphokine-activated killer cells, LAK) were isolated from spleens of RAG1^−/−^ mice by negative selection using BD Imag Biotinylated Mouse NK Cell Enrichment Cocktail (#51-9002523) and Streptavidin Particles Plus – DM (#51-9000810) as per manufacturer's instructions. Enriched NK cells were cultivated in LAK medium (MEM alpha supplemented with FBS, penicillin, streptomycin, glutamine, β-mercaptoethanol, and recombinant human IL2 (1,000 IU/mL; BioLegend, # 589108) for 7 days prior to use in killing assays.

### Mouse Studies and PB Collection

All procedures were conducted under Institutional Animal Care and Use Committee–approved protocols. Nude (Jax #00785) or RAG2^−/−^ were used for xenograft studies (6–8 weeks of age, 10 mice per cohort unless otherwise noted), and RAG1^−/−^ were used for isolation of lymphokine-activated killer (LAK) cells from spleen. For tumorigenesis studies, mice were fed drinking water containing dox (1 mg/mL, Bio-World #40410005-2) and 2.5% sucrose for 5–7 days before xenografting. Mice were subcutaneously xenografted in the hind flank with USP6/A673 cells (2.5E6) resuspended in 50% Matrigel (Corning #356234) in PBS. For the bilateral xenografting experiment to test abscopal response, parental A673 cells (2.5E6) were xenografted subcutaneously into the contralateral flank the following day. For all experiments, dox water was changed twice weekly. Tumors were measured two to three times per week using digital calipers, and volume was calculated as (length × width^2^)(π/6). Animals were sacrificed when any tumor reached terminal volume (1,500 or 2,000 mm^3^, as indicated), and tumors and spleens were harvested. Excised tumors were digested with Miltenyi Biotec tumor dissociation kit (#130-096-730); digests were subjected to flow cytometry or RNA isolation using TRIzol. qRT-PCR was performed as described previously (20, 23).

For depletion of NK cells, RAG2^−/−^ mice were injected intraperitoneally with anti-NK1.1 (Clone PK136; Bio X Cell, #BE0036) or control IgG2a antibody (300 μg diluted in 100 μL PBS), one day before xenografting USP6/A673 cells, and once per week thereafter. PB was collected from tail vein into Ethylenediaminetetraacetic acid (EDTA)-coated tubes, and red blood cells (RBC) were lysed using RBC lysis buffer (Miltenyi Biotec #130-094-183). The remaining white pellet (leukocytes) was subjected to flow cytometry analysis to quantify NK cells (NK1.1^+^ and NKp46^+^).

### *In Vitro* NK Cytotoxicity Assays

Target Ewing sarcoma cells [GFP-positive (GFP^+^)] were seeded in 22 mm wells at 5E5 cells per well; NK cells were cocultured at various ratios as indicated in presence of 200 IU/mL IL2 (BioLegend, # 589108), and the absence or presence of 2 μg/mL dox (ClonTech, #8634-1). Samples were seeded in duplicate for experiments with NK-92 cells, and in singlicate or duplicate for primary human and mouse NK cells (depending on amounts available). The following day, samples were stained with 7-Aminoactinomycin D (7AAD) and anti-CD45. Live target cells (GFP^+^ CD45^−^ 7AAD^−^) were quantified by Cytoflex S or LX flow cytometers; percent remaining live cells was calculated relative to target cells without cocultured NK cells.

Live cell imaging was performed using a Leica DMI8 inverted microscope (Leica Biosystems) equipped with VisiView software (Visitron Systems). USP6/RD-ES cells were treated overnight with or without dox. The following day, they were cocultured with NK-92 at a 1:1 ratio in 35 mm glass-bottom dishes, in phenol red-free DMEM supplemented with 10% tetracycline-free FBS in an environmental chamber set to 37°C, in the presence or absence of dox. Images were captured every 10 seconds for 1.5 hours. Dual-view live-cell imaging was performed using the Hamamatsu W-VIEW GEMINI Image splitting optics to simultaneously monitor the −dox and +dox plates.

### Flow Cytometry Analysis and qRT-PCR

Cultured cells were collected using Accutase (Stem Cell Technologies, #07920), stained with vital dye Zombie UV (BioLegend, #423108), and then stained for surface or intracellular markers as described previously (14). To detect CXCL10, cells were treated with 2 μmol/L Monensin (BioLegend, #420701) and 5 mg/mL Brefeldin A (BioLegend, #420601) for 4–6 hours to promote their intracellular retention. For analysis of intratumoral immune infiltrates, excised tumors were digested with Miltenyi Biotec tumor dissociation kit, then subjected to flow cytometry analysis as described previously (20). Data were recorded using Cytoflex LX flow cytometer and analyzed with FlowJo and GraphPad Prism software. Zombie UV (BioLegend #423108) and 7AAD (BioLegend 420202) were used for vital staining. Antibodies for flow cytometry are listed in Supplementary Table S1.

For qRT-PCR, TRIzol was used for RNA isolation, and qPCR was performed using SYBR Green (catalog no. 436765, Thermo Fisher Scientific) as described previously (20, 23).

### Statistical Analysis

All data represent results from at least three biological experiments. Statistical significance was determined for all *in vitro* flow cytometry experiments using two-sided paired *t* test, and for all *in vivo* experiments using unpaired *t* test, with *P* < 0.05 considered significant. The ratio-paired *t* test was used for all qPCR analysis. For bilateral subcutaneous xenograft model, tumor volumes ± dox were compared using nonparametric Kolmogorov–Smirnov *t* test. Kaplan–Meier curves for *in vivo* studies were analyzed by log-rank (Mantel–Cox) test. Data sharing is not applicable to this article as no data were created or analyzed in this study.

### Data Availability

The data generated in this study are available upon request from the corresponding author.

## Results

### NK Cells are Required for Suppression of Ewing Sarcoma Tumor Growth by USP6

**FIGURE 1:**
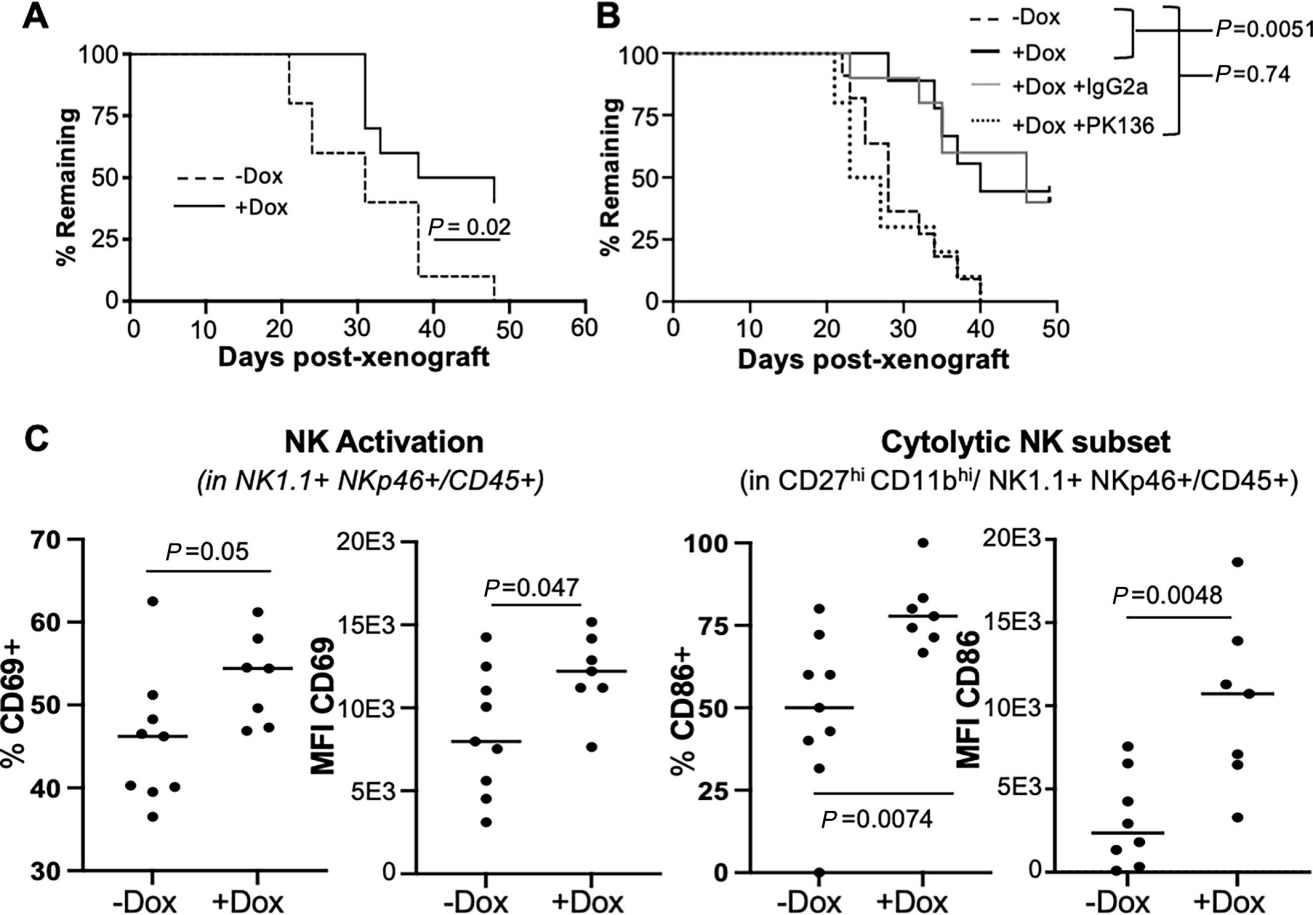
NK cells are required for inhibition of Ewing sarcoma tumor growth by USP6. **A,** RAG2^−/−^ mice were subcutaneously xenografted with USP6/A673 cells, treated with or without dox water, and tumor size recorded until a terminal volume of 2,000 mm^3^ was reached (*n* = 10 per cohort). The percent of mice remaining whose tumors have yet to reach terminal volume was plotted. **B,** RAG2^−/−^ mice were injected intraperitoneally with the NK cell–depleting antibody anti-NK1.1 (PK136) or control IgG2a, then xenografted with USP6/A673 the next day and monitored as in A (*n* = 10 per cohort). **C,** Tumors were excised, digested, and analyzed by flow cytometry. Surface levels of CD69 (activation marker) and CD86 (cytotoxicity marker) were quantified in the indicated populations. MFI, mean fluorescence intensity (−dox *n* = 9, +dox *n* = 7).

We previously demonstrated that USP6 inhibits growth of Ewing sarcoma tumors when xenografted into nude mice, but not NSG mice (20). The presence of a functional innate immune system in nude mice is the key difference between these strains, implicating its role in mediating tumor suppression. USP6’s tumor-inhibitory function was confirmed in a distinct mouse strain with intact innate immunity, RAG2^−/−^ (Fig. 1A). dox-inducible expression of USP6 in the A673 patient-derived Ewing sarcoma cell line caused a significant delay in the time required for tumors to reach terminal volume. Furthermore, only 60% of USP6-expressing tumors reached terminal volume, in contrast to 100% of those without USP6.

Given that NK cells are the main cytolytic immune lineage present in RAG2^−/−^ mice, we tested their requirement using the NK cell-neutralizing anti-NK1.1 (PK136) antibody (24). Efficient and specific NK cell depletion was confirmed through flow cytometric analysis of PB (Supplementary Fig. S1A and S1B). Mice were administered PK136 or IgG2a control antibody one day prior to xenografting of USP6/A673 cells, and weekly days thereafter (Supplementary Fig. S1C and S1D). Depletion of NK cells reversed the tumor-inhibitory effects of USP6 (Fig. 1B; Supplementary Fig. S1E). In mice depleted of NK cells, USP6-expressing tumors grew as rapidly as those lacking USP6. In contrast, USP6 retained the ability to suppress tumor growth in mice treated with control IgG2a. These results reveal that NK cells play a critical role in suppression of Ewing sarcoma tumorigenesis by USP6.

We next examined USP6 effects on NK cells within the TME. Flow cytometric analysis of tumor digests demonstrated that in RAG2^−/−^ mice, USP6 did not affect the overall abundance of immune cells (CD45^+^) or tumor-infiltrating NK cells (TINK; defined as NK1.1^+^NKp46^+^), or NK subpopulations (as defined by CD27/CD11b levels; refs. 25–27; Supplementary Fig. S2A and S2B). However, USP6 induced TINK activation, as monitored by surface levels of CD69 (Fig. 1C). USP6 also elicited a significant increase in surface expression of CD86, a marker of high cytolytic activity, specifically in the CD27^hi^/CD11b^hi^ cytolytic NK cell subpopulation (ref. 28; Fig. 1C). We also observed a trend toward increased surface expression of CD25 (IL2 receptor α chain, a marker for proliferative potential) in NK cells in the dox-treated tumors, although this did not reach statistical significance (Supplementary Fig. S2C). In sum, these results indicate that USP6 is sufficient to drive intratumoral activation of NK cells in Ewing sarcoma *in vivo*, and that NK cells are required for suppression of tumor growth by USP6.

### USP6 Expression in Ewing Sarcoma Directly Activates Primary Mouse and Human NK Cells

**FIGURE 2:**
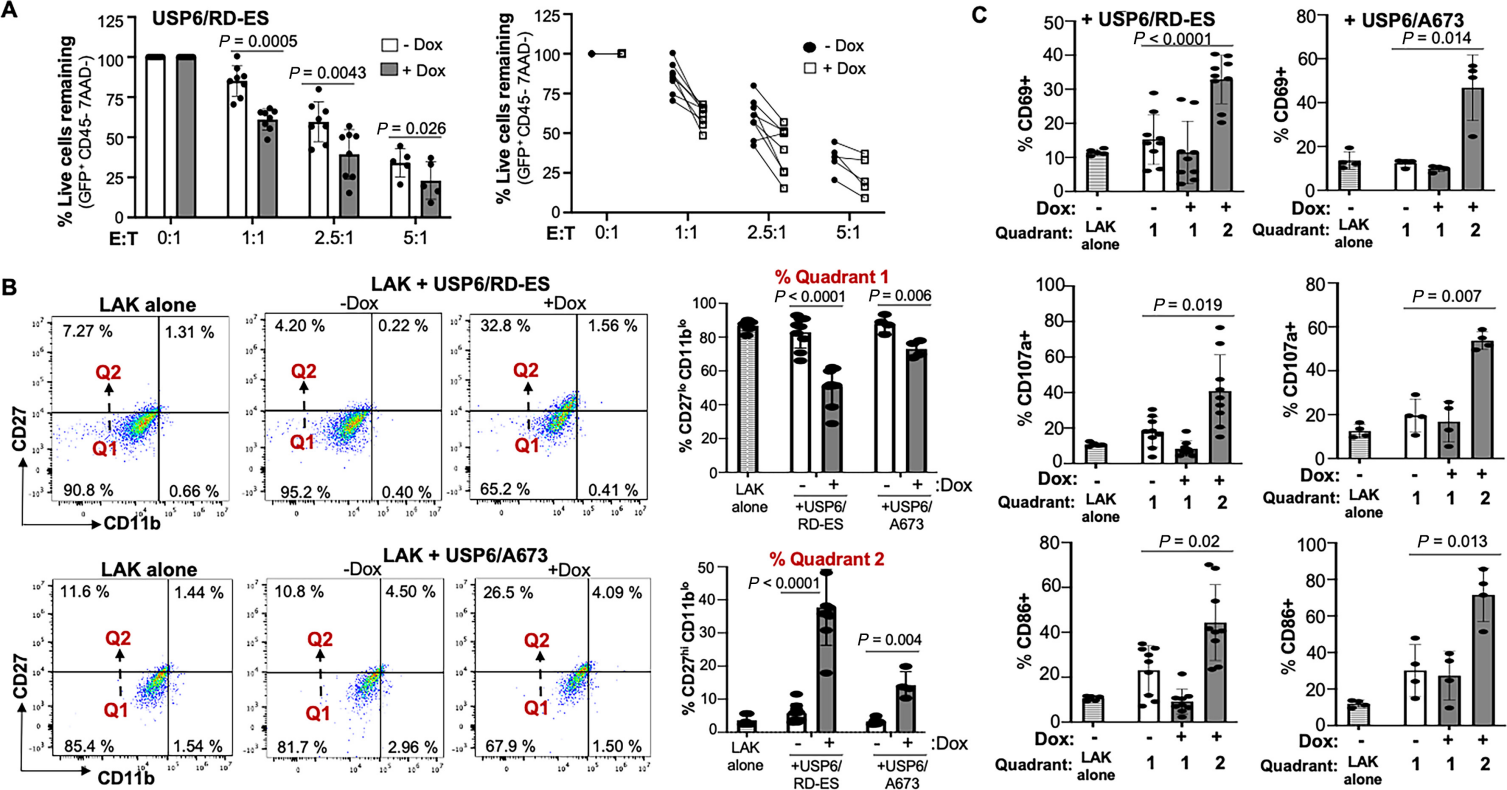
USP6 expression in Ewing sarcoma (ES) cells stimulates cytolytic activity of primary mouse NK cells. **A,** USP6/RD-ES cells were cocultured with primary mouse LAKs at the indicated E:T ratios for 24 hours, and percent remaining live Ewing sarcoma cells (GFP^+^/7AAD^−^) was quantified by flow cytometry. Right plot, alternate depiction of data where the paired −dox and +dox symbols represent results from a common LAKs preparation. **B,** CD27 and CD11b surface levels in LAKs cultured alone, or together with USP6/RD-ES or USP6/A673 cells. Percent of cells in Quadrants 1/2 (Q1/Q2) is quantified. **C,** Markers for activation (CD69), degranulation (CD107a), and cytotoxicity (CD86) were quantified in Q1/Q2 populations. Data are from four independent LAK preparations (each isolated from combined spleens of 3 mice), with each sample analyzed in technical duplicate. E:T ratio was 1:1 for USP6/RD-ES, and 2.5:1 for USP6/A673.

The activation of NK cells in USP6-expressing Ewing sarcoma tumors *in vivo* prompted us to examine whether USP6 could directly stimulate cytolytic activity of murine NK cells *in vitro*. Primary NK cells were isolated from spleens of RAG1^−/−^ mice, and expanded in high concentrations of IL2 for 1 week to generate LAKs. Cytolytic potential of LAK preparations was confirmed against YAC1 lymphoma cells as a control (Supplementary Fig. S3A). Validated LAKs were cocultured at various effector:target (E:T) ratios with two independent Ewing sarcoma cell lines expressing USP6 in a dox-inducible manner, USP6/A673 and USP6/RD-ES. After 24 hours of coculture, the percentage of remaining live Ewing sarcoma cells (GFP^+^ CD45^−^ 7AAD^−^) was quantified by flow cytometry (Supplementary Fig. S3B). LAKs were capable of killing RD-ES and A673 cells in the absence of dox, in a dose-dependent manner. However, in both cell lines, at all E:T ratios, target cell killing was enhanced in the presence of dox (Fig. 2A; Supplementary Fig. S3C).

Interestingly, not only did USP6 enhance cytolytic activity, but it also affected surface levels of the key maturation marker, CD27. Increased CD27 levels are associated with enhanced NK cell effector function, with CD27^low^/CD11b^low^ NK cells being immature/cytokine-producing, and CD27^high^/CD11b^low^ considered intermediate/cytokine-producing and possessing greater cytolytic potential (26, 27). When cocultured with USP6/Ewing sarcoma cells in the absence of dox, LAKs expressed low levels of CD27 and CD11b, comparable with LAKs alone (Quadrants 1 in Fig. 2B). However, upon addition of dox, there was a reproducibly discernable increase in the expression levels of CD27 on LAKs (Quadrant 2 in Fig. 2B). The effects were more pronounced upon coculture of LAKs with USP6/RD-ES than with USP6/A673. Notably, the LAK subpopulation that emerged upon USP6 expression (Quadrant 2) exhibited increased markers of activation (CD69), degranulation (CD107a), and cytotoxicity (CD86; ref. 28; Fig. 2C).

**FIGURE 3:**
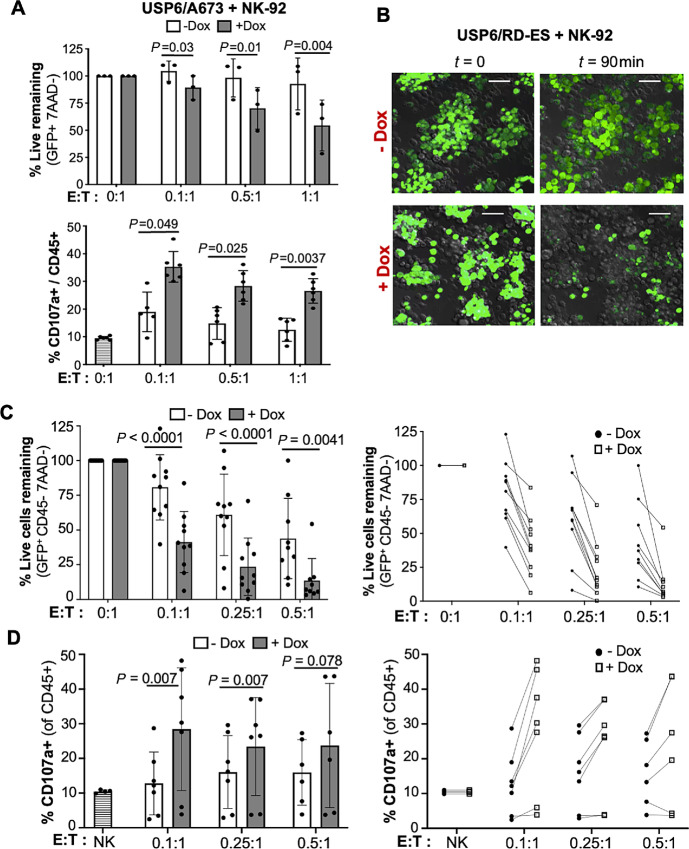
USP6 expression in Ewing sarcoma (ES) stimulates cytolytic activity of immortalized and primary human NK cells. **A,** USP6/A673 were cocultured with immortalized NK cell line NK-92 at the indicated E:T ratios, and percent remaining live Ewing sarcoma cells was quantified (*n* = 3). **B,** Live cell imaging of cocultured NK-92 (gray) and USP6/RD-ES (green) in the absence (top) or presence (bottom) dox. Images from start (*t* = 0) and end (*t* = 90 minutes) timepoints are shown; scale bar, 50 μm. Note that the endpoint frames were digitally enhanced to facilitate visualization of photobleached but live GFP^+^ cells remaining. Timelapse imaging is provided in Supplementary Video S1. **C,** USP6/RD-ES were cocultured with primary human NK cells, and percent remaining live Ewing sarcoma cells was quantified. Data represent analysis from five independent experiments using 5 distinct human donors. **D,** Degranulation in primary human NK cells was monitored by CD107a surface staining (data from 5 independent donors). In C and D, right plots depict paired −dox and +dox samples treated with NK cells from a common donor.

We sought to confirm that USP6 can activate human NK cells, given that cytolytic activity of murine NK cells against human target cells could be artifactually elevated because of loss of inhibitory signaling cross-species (29–31). Studies were first performed using the immortalized human NK cell line, NK-92. NK-92 cells displayed increased cytotoxicity toward A673 upon USP6 expression (Fig. 3A), similarly to what we reported previously for RD-ES cells (20). Increased surface expression of CD107a in NK-92 in the presence of dox confirmed that USP6 stimulates degranulation (Fig. 3A). Furthermore, live cell imaging of cocultured NK-92 and USP6/RD-ES revealed a dramatic increase in target cell killing in the presence of dox, as evidenced by abrupt destruction of GFP^+^ target cells (Fig. 3B; Supplementary Video S1).

Because NK-92 exhibit high baseline cytolytic activity due to absence of most NK cell inhibitory receptors (32, 33), we also validated USP6 effects on primary human NK cells. Killing assays were performed using NK cells isolated from PB of healthy donors, and cocultured with USP6/RD-ES or USP6/A673 cells. As shown (Fig. 3C and D; Supplementary Fig. S3D and S3E), USP6 enhanced killing of both RD-ES and A673 at all E:T ratios. Furthermore, USP6 enhanced degranulation by primary human NK cells as measured by surface CD107a expression.

### USP6 Upregulates Surface Expression of NK-activating Ligands on Ewing Sarcoma Cells *In Vitro* and *In Vivo*

**FIGURE 4:**
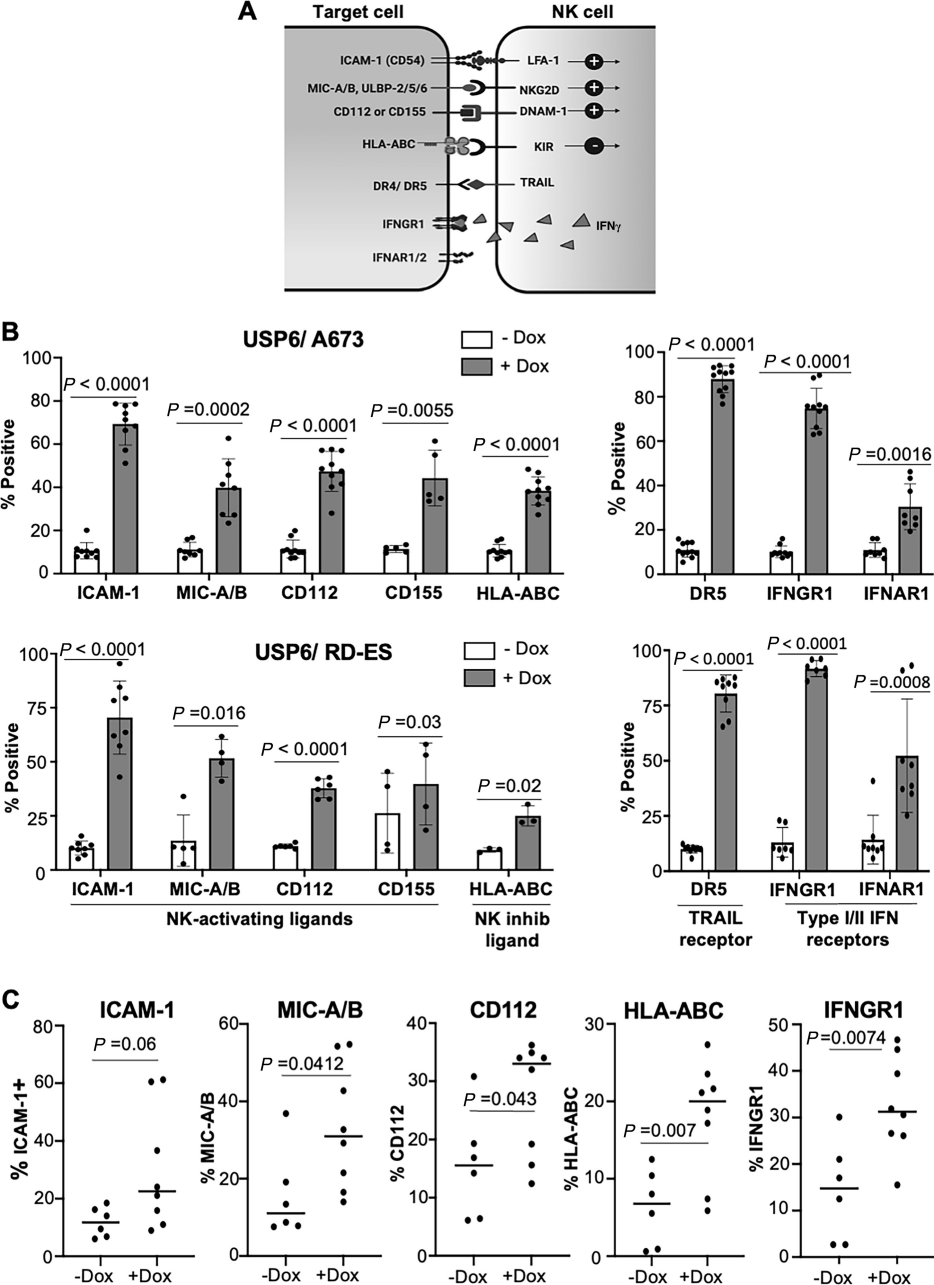
USP6 upregulates NK cell-activating ligands on Ewing sarcoma cells *in vitro* and *in vivo.***A,** Activating and inhibitory receptors on NK cells, and their cognate ligands on target cells. **B,** USP6/A673 or USP6/RD-ES cells were treated with or without dox, and surface levels of the indicated markers was quantified by flow cytometry (*n* ≥ 3 experiments per marker). **C,** USP6/A673 xenografts from RAG2^−/−^ mice were digested and subjected to flow cytometry to detect surface levels of the indicated markers (−dox *n* = 6, +dox *n* = 8).

NK cell activity is regulated by an array of germline-encoded, non–antigen-specific receptors that recognize target cells without prior sensitization and independently of MHCI (29–31). Cytolytic activity is dictated by the balance of signaling from NK-activating and inhibitory receptors, which are controlled by cognate ligands on target cells. To elucidate the mechanism by which USP6 stimulates NK cell activity, we examined its effect on expression of NK ligands (Fig. 4A). Strikingly, in both RD-ES and A673 cells, USP6 was sufficient to increase surface levels of multiple NK cell-activating factors, including ICAM-1 (CD54), which binds to the integrin LFA-1 on NK and T cells to mediate immune synapse formation and polarization of cytolytic granules to the site of cell contact (ref. 34; Fig. 4B). In addition, USP6 increased surface levels of MIC-A/B, CD112, and CD155, which are ligands for the NK cell activating receptors NKG2D and DNAM-1, respectively, as well as the inhibitory ligand HLA-ABC (Fig. 4A and B). USP6 expression did not significantly or reproducibly affect mRNA levels of any of these factors (Supplementary Fig. S4A). The effects were selective, as USP6 had no effect on surface levels of other NK cell regulatory ligands (including ULBP2/5/6, CD48, and HLA-E), or on NK receptors in NK-92 cells. Together, these results indicate that USP6 expression in Ewing sarcoma cells directly activates NK cells by enhancing both immune synapse formation (via ICAM-1) as well as degranulation via multiple NK cell-activating ligands.

In addition to killing target cells through release of cytolytic granules, NK cells can also induce death in susceptible cell types through production of TRAIL and IFNγ (29–31). In both RD-ES and A673 cells, USP6 induced dramatic surface upregulation of the TRAIL receptor, DR5 (Fig. 4B). Surface levels of type I and II IFN receptors (IFNAR1 and IFNGR1, respectively) were also significantly increased by USP6, as we reported previously (Fig. 4B; ref. 20). Thus, not only does USP6 enhance the cytolytic function of NK cells, but it may also enhance sensitivity of Ewing sarcoma cells to killing through TRAIL, as supported by our previous studies (23).

To assess whether USP6 could also upregulate these factors *in vivo*, flow cytometry was performed on digested USP6/A673 xenografts. Strikingly, we confirmed that USP6 was capable of increasing surface expression of NK cell activating ligands, HLA-ABC, and IFNGR1 in A673 tumors *in vivo* (Fig. 4C; Supplementary Fig. S4B).

### USP6/Ewing Sarcoma and NK Cells Participate in a Paracrine Feedforward Loop

**FIGURE 5:**
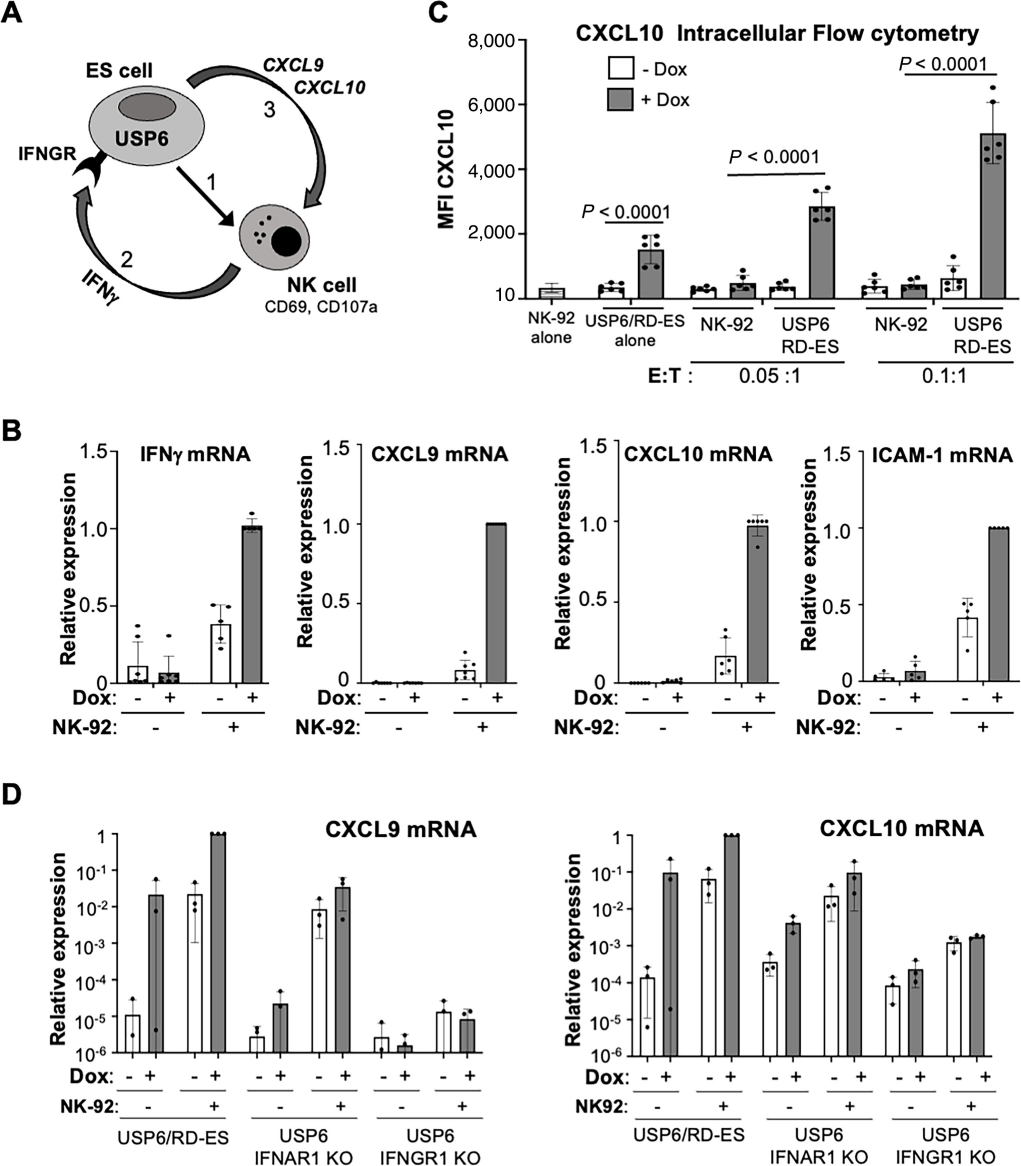
USP6/Ewing sarcoma (ES) and NK cells participate in a paracrine feedforward loop mediated through IFNGR signaling in Ewing sarcoma cells. **A,** Diagram of proposed paracrine feedforward loop between USP6-expressing Ewing sarcoma cells and NK cells: (1) USP6/Ewing sarcoma cells activate NK cells; (2) activated NK cells secrete IFNγ, which feeds back on USP6/Ewing Sarcoma cells which have elevated surface levels of IFNGR; (3) IFNγ-inducible chemokines CXCL9/CXCL10 are synergistically induced in USP6-expressing cells, stimulating further NK cell recruitment, maturation, and activation. USP6/RD-ES were treated ± dox, in the absence or presence of NK-92 (0.1:1 E:T ratio). Samples were subjected to qRT-PCR (*n* ≥ 3; **B**), or intracellular flow cytometry to detect CXCL10 (*n* = 3; **C**). **D,** NK-92 were cocultured with USP6/RD-ES (either WT, or CRISPR-deleted IFNAR1 or IFNGR1 derivatives) at 0.1:1 ratio overnight, then subjected to qRT-PCR. CXCL9/CXCL10 mRNA levels were expressed relative to those in USP6/wild type (WT) RD-ES cells cocultured with NK-92 in the presence.

We previously reported that USP6 is sufficient to induce type I and type II IFN response gene signatures, which includes the immunostimulatory chemokines *CXCL9* and *CXCL10* (20). Furthermore, USP6 renders Ewing sarcoma cells hyperresponsive to IFNs, such that IFN response genes are synergistically induced by ectopic IFNγ in Ewing sarcoma cells expressing USP6. This led us to speculate that a paracrine feedforward loop might be generated between USP6/Ewing sarcoma and NK cells (see model, Fig. 5A), wherein NK cells activated by USP6 produce elevated levels of *IFNγ*, which feeds back on the USP6/Ewing sarcoma cells to induce synergistic expression of IFN response genes such as *CXCL9*/*CXCL10* due to their elevated expression of IFNGR1 and Jak1 (20). *CXCL9*/*CXCL10* would then trigger further recruitment, activation, and maturation of NK cells (35, 36). To test this, RT-PCR was performed on cocultured USP6/Ewing sarcoma and NK-92 cells. Induction of IFNγ was indeed enhanced in the presence of dox (Fig. 5B; Supplementary Fig. S5A; ref. 20), consistent with enhanced activation of NK-92 by USP6. As predicted, USP6 amplified induction of *CXCL9*, *CXCL10*, and *ICAM-1* upon coculture with NK-92 cells (Fig. 5B; Supplementary Fig. S5A).

To further test this feedforward model, we sought to confirm whether CXCL9/CXCL10 were produced by the USP6/RD-ES cells, because NK cells are also capable of producing these chemokines (29, 34–36). Intracellular flow cytometry confirmed that the USP6/Ewing sarcoma cells, and not the NK-92, were the predominant source of CXCL10 (Fig. 5C; Supplementary Fig. S5B). Next, to validate whether IFNγ mediates induction of CXCL9/CXCL10, we depleted IFNGR1 or IFNAR1 from USP6/RD-ES cells (Supplementary Fig. S5C; ref. 20). As shown in Fig. 5D, synergistic induction of CXCL9/CXCL10 was completely ablated by IFNGR1 depletion, and partly inhibited by IFNAR1 depletion, indicating that Ewing sarcoma–intrinsic signaling through both pathways contributes to this feedforward loop. Together, these data strongly support the paracrine feedforward model presented in Fig. 5A.

### USP6 Induces Systemic Activation and Maturation of NK Cells

**FIGURE 6:**
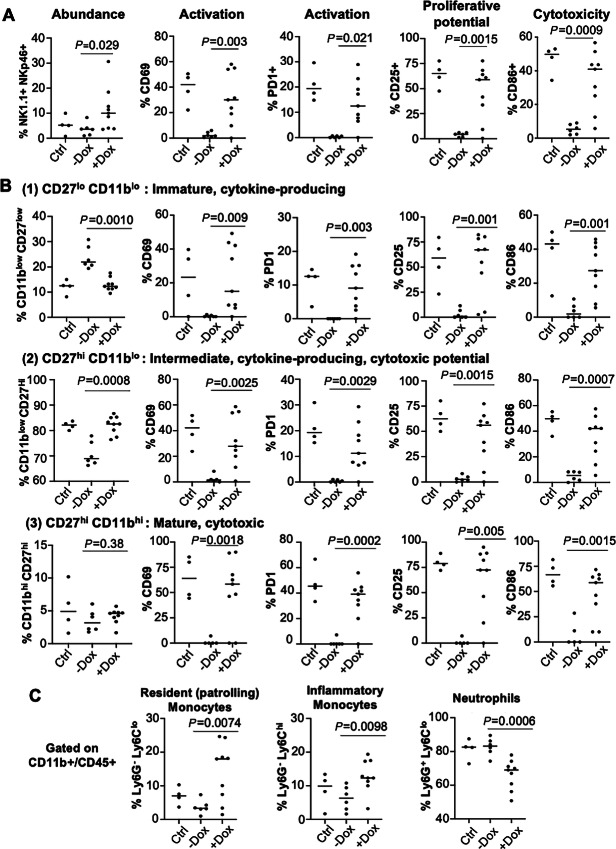
Intratumoral USP6 expression induces systemic activation of NK and myeloid cells. RAG2^−/−^ mice were xenografted with USP6/A673 and treated with or without dox, or not xenografted (Control, Ctrl). PB was collected approximately 3 weeks later, and subjected to flow cytometry. Abundance, early/late activation, proliferative potential, and cytotoxic activity were assessed using the indicated markers in the entire NK population (NK1.1^+^/NKp46^+^; **A**), and in NK cell subpopulations as defined by levels of CD27 and CD11b (**B**; see also Supplementary Fig. S6 for definition of subsets). **C,** Abundance of myeloid populations (among the CD11b^+^/CD45^+^ cells) as defined by the indicated markers (Ctrl *n* = 4, −dox *n* = 6, +dox *n* = 9).

Given the robust NK cell activation and paracrine feedforward loop induced by USP6 intratumorally, we speculated that USP6 might trigger systemic immune activation *in vivo*. To test this, PB was collected from RAG2^−/−^ mice that were subcutaneously xenografted with USP6/A673 cells, and NK and myeloid lineages were analyzed by flow cytometry (Fig. 6; Supplementary Fig. S6). Mice bearing USP6-expressing tumors had significantly increased abundance of circulating NK cells (NK1.1^+^ NKp46^+^) when compared with non-xenografted mice or USP6/A673 xenografted mice without dox treatment (Fig. 6A). Control A673 tumors (in mice without dox treatment) severely inhibited the function of NK cells in PB, as indicated by the suppression of markers for activation (CD69 and PD1), proliferative capacity (CD25), and cytotoxicity (CD86; Fig. 6A; Supplementary Fig. S6B). Remarkably, expression of USP6 intratumorally restored the expression of all of these markers in circulating NK cells.

We also examined the maturation state of circulating NK cells. As discussed above, levels of CD11b and CD27 define maturational and functional subsets of NK cells (Fig. 6B; Supplementary Fig. S6C and S6D). In the absence of USP6, A673 xenografts shifted the relative abundance of the cytokine-producing subpopulations from intermediate (CD27^hi^ CD11b^low^) to immature (CD27^low^ CD11b^low^) state (Fig. 6B). Strikingly, USP6 was sufficient to restore the levels of intermediate cytokine-producing NK cells back to those observed in non-xenografted mice (Fig. 6B). Furthermore, in all NK cell subpopulations, A673 xenografts dramatically inhibited NK cell activation/degranulation (CD69/CD107a), proliferative capacity (CD25), and cytotoxicity (CD86; Fig. 6B). Again, USP6 restored all of these parameters to control levels in non-xenografted mice. Thus, not only does USP6 activate NK cells intratumorally, but it also has broad effects on NK cells systemically, increasing their abundance, maturation, cytolytic capability, and proliferative potential in circulation.

Given its striking effects on circulating NK cells, we surmised that USP6 might also impact myeloid cells systemically. We found that intratumoral USP6 expression significantly increased the relative abundances of immune-stimulatory nonclassical (Ly6G^−^Ly6C^low^) monocytes and classical (Ly6G^−^Ly6C^hi^) monocytes, but reduced levels of protumorigenic neutrophils (Ly6G^+^Ly6C^−^) in PB (Fig. 6C; Supplementary Fig. S7). Together, these results reveal that intratumoral expression of USP6 in Ewing sarcoma has broad effects on systemic innate immune activity.

### USP6 Elicits an Abscopal Response and Immune Activation within Distal Tumors

**FIGURE 7:**
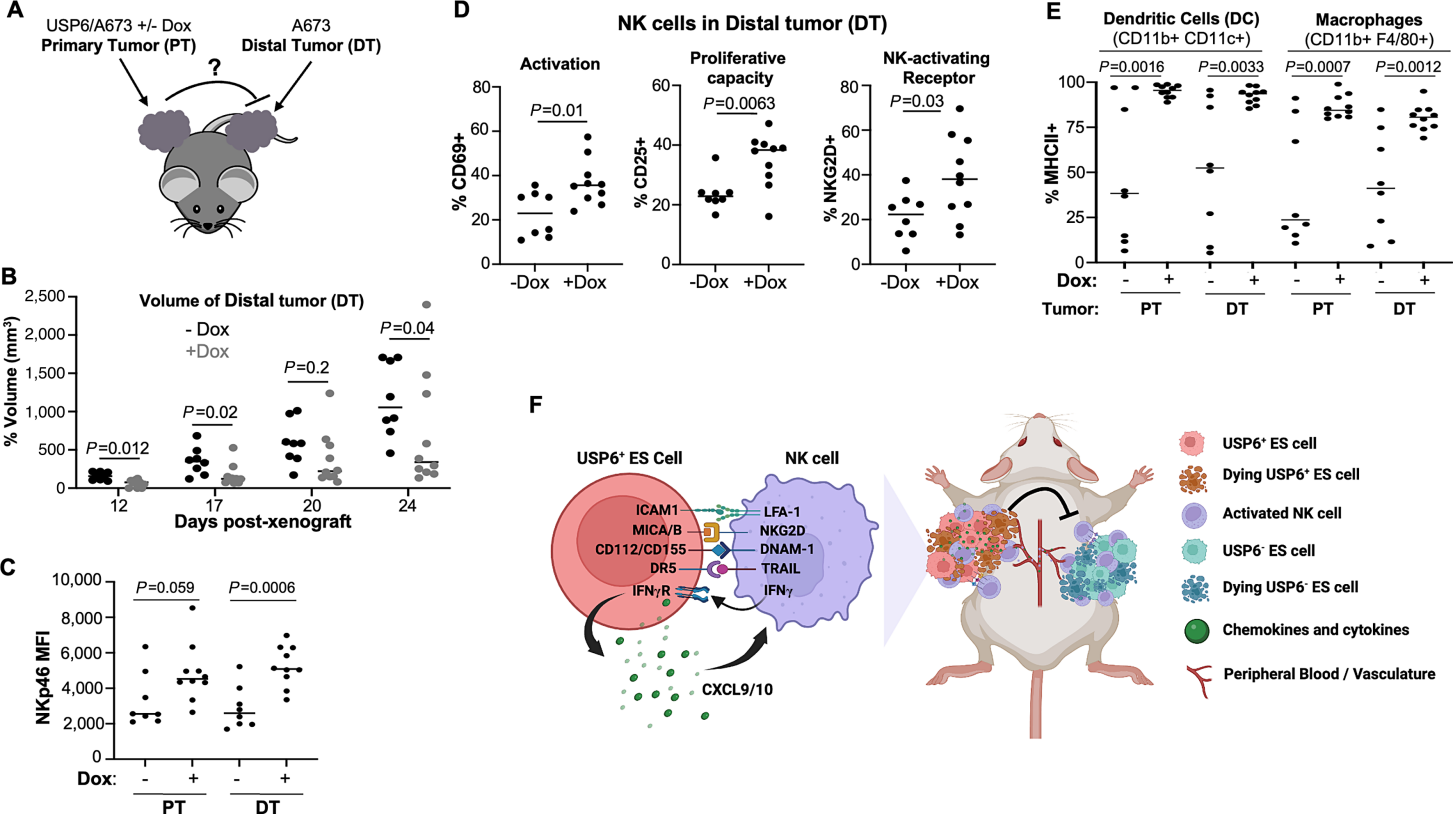
USP6 elicits an abscopal response with infiltration and activation of NK cells in DTs. **A,** Experimental set-up to test abscopal response using bilateral subcutaneous xenografts (*n* = 10 per cohort). **B,** Volume of DTs in the absence or presence of dox. Tumors were digested, and subjected to flow cytometry to quantify: expression levels of NKp46 in the NK1.1^+^/F4/80^−^/Ly6G^−^ NK cell population in PTs and DTs (**C**); activation, proliferative capacity, and NKG2D expression in NKs in DTs (**D**); and activation of DCs and macrophages in PTs and DTs, as monitored by MHCII levels (*n* = 8 for –dox, and *n* = 10 for +dox; **E**). **F,** Summary and model of immunostimulatory and antitumorigenic functions of USP6 in Ewing sarcoma. Left, mechanism of NK cell activation and paracrine immunostimulatory feedforward loop; right, depiction of abscopal response (diagram created using Biorender.com).

The ability of USP6 to induce systemic immune activation led us to explore whether it can elicit an abscopal response [i.e., growth suppression of distal tumors (DT) not expressing USP6]. To test this, nude mice were subcutaneously xenografted bilaterally (Fig. 7A): USP6/A673 cells were injected into one flank (PT), and parental A673 cells were xenografted in the contralateral flank (DT). Remarkably growth of DTs was strongly inhibited (Fig. 7B). Even more strikingly, USP6 significantly increased surface levels of the NK cell-activating receptor NKp46^+^ in DTs (Fig. 7C; Supplementary Fig. S8A). Furthermore, in mice bearing USP6^+^ PTs, NK cells in DTs exhibited enhanced activation (CD69^+^) and proliferative capacity (CD25^+^), and increased expression of the NK-activating receptor NKG2D (Fig. 7D; Supplementary Fig. S8A). USP6 also exerted effects on myeloid lineages, inducing dramatic upregulation of MHCII in dendritic cells (DC) and macrophages in both primary and DTs (Fig. 7E; Supplementary Fig. S8B).

We attempted to test the abscopal response using additional Ewing sarcoma cell lines (RD-ES and TC-71); however, this was not technically feasible. While A673 have a relatively narrow timeframe for both tumor latency and time required to reach terminal volume, the range for these parameters was much broader for RD-ES and TC71. Compounded by bilateral xenografting, it was not possible to draw statistically meaningful comparisons between −dox and +dox cohorts.

## Discussion

Our study provides further insight into the mechanism by which USP6 modulates the immune TME in Ewing sarcoma to suppress tumorigenesis. We previously reported that USP6 induces intratumoral infiltration and activation of multiple innate immune lineages in Ewing sarcoma xenografts in nude mice. Here, we demonstrate an essential role for NK cells in USP6-mediated tumor suppression. USP6 directly stimulates cytolytic activity of primary human and murine NK cells, and functions by upregulating surface expression of multiple NK cell-activating ligands and receptors for apoptotic factors in Ewing sarcoma cells (see summary in Fig. 7F, left). Upon activation, NK cells secrete IFNγ, which feeds back on USP6-expressing Ewing sarcoma cells to synergistically induce CXCL9/CXCL10. We posit that *in vivo*, this feedforward loop creates a highly immunostimulatory, chemokine-rich TME, which leads to further recruitment and activation of not only NK cells, but also CD8^+^ T lymphocytes, macrophages, and DCs, as is seen in Ewing sarcoma patient samples with high USP6 expression (20). Strikingly, USP6’s immunostimulatory effect is not confined to tumors, as systemic activation of NK and myeloid cells is also induced (Fig. 6). Consistent with systemic immune activation, USP6 triggers an abscopal response, inhibiting growth of distal non–USP6-expressing Ewing sarcoma tumors, and inducing infiltration and activation of NK cells, DCs, and macrophages within them (Fig. 7F, right). It is tempting to speculate that USP6’s ability to induce systemic immune activation and an abscopal response underlies the association of high USP6 expression with significantly improved overall survival of patients with Ewing sarcoma (20).

USP6 remains the only cancer cell–intrinsic factor, to our knowledge, demonstrated to modulate the immune landscape in Ewing sarcoma. Study and manipulation of the Ewing sarcoma immune TME has been significantly hampered by the lack of immunocompetent mouse models for the disease (37). However, our studies underscore the utility of partially immunocompetent nude and RAG2^−/−^ strains for study of the Ewing sarcoma immune landscape, as key elements of human disease can be captured in these models. First, innate lineages are the major component of immune infiltrates in patients with Ewing sarcoma (10, 19, 38), and nude/RAG2^−/−^ mice retain functional innate immunity. Second, cytolytic activity of circulating NK cells is reduced in patients with Ewing sarcoma compared with healthy age-matched donors (39), and this is reproduced in Ewing sarcoma xenografted RAG2^−/−^ mice. Third, multivariate Cox regression analysis identified intratumoral activated NK cells as the single most significant independent prognostic immune lineage for overall survival in patients with Ewing sarcoma, above even T cells (19). Our results similarly point to a pivotal role for NK cells in Ewing sarcoma tumor growth, with depletion of NK cells completely abolishing the tumor-suppressive effects of USP6.

A reasonable concern with xenograft models is that mouse NK cells have artifactually elevated cytolytic activity against xenografted human Ewing sarcoma cells because they lack “self” MHCI to suppress effector function (25, 29). Indeed, mouse LAKs are capable of killing control RD-ES and A673 cells (Fig. 2A; Supplementary Fig. S3C, see −dox samples). However, our data clearly demonstrate that USP6 increases degranulation, activation, and cytolytic function of mouse NK cells above and beyond this baseline level, both directly *in vitro* (Fig. 2; Supplementary Fig. S3) and *in vivo* (Figs. 1, 6, 7; ref. 20). Furthermore, in cocultured primary mouse LAKs and Ewing sarcoma cells, USP6 promotes LAKs maturation (from CD27^low^CD11bl^ow^ to CD27^high^CD11b^low^). Finally, we show that USP6 is capable of activating human NK cells and identify the underlying mechanism, indicating that activation of mouse NK cells is not simply a cross-species artifact. Thus, although due caution must be applied, we aver that nude and RAG2^−/−^ mice can provide useful insights into the role and regulation of innate immunity (particularly NK cells) in Ewing sarcoma pathogenesis.

A remarkable aspect of USP6’s immunomodulatory function is its ability to ignite immune activation systemically. As mentioned, effector function of circulating NK cells is reduced in patients with Ewing sarcoma compared with healthy controls (39). We validated that in RAG2^−/−^ mice bearing Ewing sarcoma xenografts, activated effector NK cells in PB were significantly depressed. Strikingly, USP6 reversed this, restoring both the abundance and activation of circulating NK cells back to levels in control non–tumor-bearing mice (Fig. 6A and B). This is particularly exciting given that peripheral NK cells are known to play a critical role in controlling metastasis, because they can kill targets in circulation (unlike CD8^+^ T cells). USP6 also increased levels of peripheral monocytes (patrolling and inflammatory), and decreased levels of neutrophils (Fig. 6C). USP6 was able to induce an abscopal response against DTs, and future efforts will be aimed at determining the contributions of soluble and cellular factors, including NK cells and myeloid lineages. In this context, it is interesting to note that patrolling monocytes can promote NK cell activation (40, 41), while neutrophils can shield cancer cells from clearance by NK cells (42, 43).

Immunotherapy has not been successfully utilized in Ewing sarcoma, and our results underscore the great potential of USP6 for advancing novel approaches. For example, recent studies revealed that patients with relapsed Ewing sarcoma actually have circulating effector CD8^+^ T cells but they fail to infiltrate tumors, leading the authors to speculate that introduction of CXCL10-elevating agents into metastatic lesions might be an effective means to drive homing of the effector T cells (38). Similarly, adoptive cell transfer of NK cells was shown to curb growth of primary Ewing sarcoma tumors and lung metastases in mouse models, but efficacy was limited because of poor homing (15–18). These results underscore the need to induce intratumoral production of CXCL10 to achieve sufficient recruitment of cytolytic lineages (10, 16–18, 38, 44). In addition, MHCI expression is frequently depressed or absent in Ewing sarcoma tumors, and it has been proposed that increasing surface MHCI could be a means of stimulating CD8^+^ T cells and promoting ICI responsiveness in patients with Ewing sarcoma (10). Efforts are currently underway to elicit these responses through intratumoral delivery of *USP6* mRNA encapsulated in lipid nanoparticles (LNP).

On a final note, we posit that due to USP6’s ability to promote a hot TME and broad innate immune activation (20), it holds promise not only as a monotherapy for Ewing sarcoma (44, 45), but also for other malignancies and in combination therapy. A hot TME portends improved overall survival across many cancers (21). In addition, USP6 LNPs could serve as an ideal adjuvant for ICIs, whose efficacy is predicated on a hot TME (21, 46). For chimeric antigen receptor T cells, efficacy is severely limited in solid cancers, where tumor infiltration and the immunosuppressive TME are critical hurdles. USP6 could aid in overcoming these challenges through its ability to engender an IFN response signature, and a chemokine-rich, immunologically hospitable TME (20). Finally, USP6’s ability to ignite NK cell activation (both locally and systemically) adds another dimension to its antitumorigenic capabilities.

## Supplementary Material

Supplementary Table S1 Flow antibodies used

Supplementary Figure S1 NK depletion confirmation

Supplementary Figure S2 Gating for NK populations and intratumoral quantification

Supplementary Figure S3 Primary mouse and human NK killing assays

Supplementary Figure S4 RT-qPCR of surface markers induced by USP6

Supplementary Figure S5 Paracrine feedforward loop between NK and USP6-A673 cells

Supplementary Figure S6 Gating strategy for NK cells in peripheral blood

Supplementary Figure S7 Gating strategy for myeloid lineages in peripheral blood

Supplementary Figure S8 Gating strategy for immune lineages in abscopal response experiment

Supplementary Video S1 Live cell imaging of NK92 killing USP6/RD-ES cells

## References

[1] Grunewald TGP , Cidre-Aranaz F, Surdez D, Tomazou EM, de Alava E, Kovar H, . Ewing sarcoma. Nat Rev Dis Primers 2018;4:5. 29977059 10.1038/s41572-018-0003-x

[2] Lessnick SL , Ladanyi M. Molecular pathogenesis of Ewing sarcoma: new therapeutic and transcriptional targets. Annu Rev Pathol 2012;7:145–59. 21942527 10.1146/annurev-pathol-011110-130237 PMC3555146

[3] Riggi N , Suva ML, Stamenkovic I. Ewing's sarcoma. N Engl J Med 2021;384:154–64. 33497548 10.1056/NEJMra2028910

[4] Gangwal K , Sankar S, Hollenhorst PC, Kinsey M, Haroldsen SC, Shah AA, . Microsatellites as EWS/FLI response elements in Ewing's sarcoma. Proc Natl Acad Sci U S A 2008;105:10149–54. 18626011 10.1073/pnas.0801073105 PMC2481306

[5] Riggi N , Knoechel B, Gillespie SM, Rheinbay E, Boulay G, Suva ML, . EWS-FLI1 utilizes divergent chromatin remodeling mechanisms to directly activate or repress enhancer elements in Ewing sarcoma. Cancer Cell 2014;26:668–81. 25453903 10.1016/j.ccell.2014.10.004 PMC4492343

[6] Gartrell J , Rodriguez-Galindo C. Ewing sarcoma: investigational mono- and combination therapies in clinical trials. Expert Opin Investig Drugs 2021;30:653–63. 10.1080/13543784.2021.1919623 33870845

[7] Morales E , Olson M, Iglesias F, Dahiya S, Luetkens T, Atanackovic D. Role of immunotherapy in Ewing sarcoma. J Immunother Cancer 2020;8:e000653. 33293354 10.1136/jitc-2020-000653 PMC7725096

[8] Hutzen B , Ghonime M, Lee J, Mardis ER, Wang R, Lee DA, . Immuno-therapeutic challenges for pediatric cancers. Mol Ther Oncolytics 2019;15:38–48. 31650024 10.1016/j.omto.2019.08.005 PMC6804520

[9] Rossig C . Cellular immunotherapy strategies for Ewing sarcoma. Immunotherapy 2014;6:611–21. 24896629 10.2217/imt.14.36

[10] Evdokimova V , Gassmann H, Radvanyi L, Burdach SEG. Current state of immunotherapy and mechanisms of immune evasion in Ewing sarcoma and osteosarcoma. Cancers 2022;15:272. 36612267 10.3390/cancers15010272 PMC9818129

[11] Berghuis D , de Hooge AS, Santos SJ, Horst D, Wiertz EJ, van Eggermond MC, . Reduced human leukocyte antigen expression in advanced-stage Ewing sarcoma: implications for immune recognition. J Pathol 2009;218:222–31. 19274709 10.1002/path.2537

[12] Yabe H , Tsukahara T, Kawaguchi S, Wada T, Torigoe T, Sato N, . Prognostic significance of HLA class I expression in Ewing's sarcoma family of tumors. J Surg Oncol 2011;103:380–5. 21400519 10.1002/jso.21829

[13] Lopez-Soto A , Gonzalez S, Smyth MJ, Galluzzi L. Control of metastasis by NK cells. Cancer Cell 2017;32:135–54. 28810142 10.1016/j.ccell.2017.06.009

[14] Zamai L , Ponti C, Mirandola P, Gobbi G, Papa S, Galeotti L, . NK cells and cancer. J Immunol 2007;178:4011–16. 17371953 10.4049/jimmunol.178.7.4011

[15] Kimpo MS , Oh B, Lee S. The role of natural killer cells as a platform for immunotherapy in pediatric cancers. Curr Oncol Rep 2019;21:93. 31502008 10.1007/s11912-019-0837-8 PMC6733832

[16] Cho D , Shook DR, Shimasaki N, Chang YH, Fujisaki H, Campana D. Cytotoxicity of activated natural killer cells against pediatric solid tumors. Clin Cancer Res 2010;16:3901–9. 20542985 10.1158/1078-0432.CCR-10-0735 PMC3168562

[17] Tong AA , Hashem H, Eid S, Allen F, Kingsley D, Huang AY. Adoptive natural killer cell therapy is effective in reducing pulmonary metastasis of Ewing sarcoma. Oncoimmunology 2017;6:e1303586. 28507811 10.1080/2162402X.2017.1303586 PMC5414867

[18] Yalcin K , Ovali E, Ozdamarlar U, Celen S, Karasu G, Yesilipek A, . NK-92 cellular therapy for pediatric relapsed/refractory Ewing sarcoma. Int Cancer Conf J 2020;9:137–40. 32582518 10.1007/s13691-020-00406-6 PMC7297928

[19] Stahl D , Gentles AJ, Thiele R, Gutgemann I. Prognostic profiling of the immune cell microenvironment in Ewing's sarcoma. Oncoimmunology 2019;8:e1674113. 31741777 10.1080/2162402X.2019.1674113 PMC6844324

[20] Henrich IC , Jain K, Young R, Quick L, Lindsay JM, Park DH, . Ubiquitin-specific protease 6 functions as a tumor suppressor in Ewing sarcoma through immune activation. Canc Res 2021;81:2171–83. 10.1158/0008-5472.CAN-20-1458 PMC8137534 33558334

[21] Gentles AJ , Newman AM, Liu CL, Bratman SV, Feng W, Kim D, . The prognostic landscape of genes and infiltrating immune cells across human cancers. Nat Med 2015;21:938–45. 26193342 10.1038/nm.3909 PMC4852857

[22] Quick L , Young R, Henrich IC, Wang X, Asmann YW, Oliveira AM, . Jak1–STAT3 signals are essential effectors of the USP6/TRE17 oncogene in tumorigenesis. Canc Res 2016;76:5337–47. 10.1158/0008-5472.CAN-15-2391 PMC5026615 27440725

[23] Henrich IC , Young R, Quick L, Oliveira AM, Chou MM. USP6 confers sensitivity to IFN-mediated apoptosis through modulation of TRAIL signaling in Ewing sarcoma. Mol Canc Res 2018;16:1834–43. 10.1158/1541-7786.MCR-18-0289 PMC6279478 30131449

[24] Wilson KA , Goding SR, Neely HR, Harris KM, Antony PA. Depletion of B220^+^NK1.1^+^ cells enhances the rejection of established melanoma by tumor-specific CD4^+^ T cells. Oncoimmunology 2015;4:e1019196. 26405570 10.1080/2162402X.2015.1019196 PMC4570124

[25] Sun JC , Lanier LL. NK cell development, homeostasis and function: parallels with CD8^+^ T cells. Nat Rev Immunol 2011;11:645–57. 21869816 10.1038/nri3044 PMC4408539

[26] Chiossone L , Chaix J, Fuseri N, Roth C, Vivier E, Walzer T. Maturation of mouse NK cells is a 4-stage developmental program. Blood 2009;113:5488–96. 19234143 10.1182/blood-2008-10-187179

[27] Hayakawa Y , Smyth MJ. CD27 dissects mature NK cells into two subsets with distinct responsiveness and migratory capacity. J Immunol 2006;176:1517–24. 16424180 10.4049/jimmunol.176.3.1517

[28] Peng Y , Luo G, Zhou J, Wang X, Hu J, Cui Y, . CD86 is an activation receptor for NK cell cytotoxicity against tumor cells. PLoS One 2013;8:e83913. 24349559 10.1371/journal.pone.0083913 PMC3859666

[29] Long EO , Kim HS, Liu D, Peterson ME, Rajagopalan S. Controlling natural killer cell responses: integration of signals for activation and inhibition. Annu Rev Immunol 2013;31:227–58. 23516982 10.1146/annurev-immunol-020711-075005 PMC3868343

[30] Morvan MG , Lanier LL. NK cells and cancer: you can teach innate cells new tricks. Nat Rev Cancer 2016;16:7–19. 26694935 10.1038/nrc.2015.5

[31] Prager I , Watzl C. Mechanisms of natural killer cell-mediated cellular cytotoxicity. J Leukoc Biol 2019;105:1319–29. 31107565 10.1002/JLB.MR0718-269R

[32] Klingemann H , Boissel L, Toneguzzo F. Natural killer cells for immunotherapy – advantages of the NK-92 cell line over blood NK cells. Front Immunol 2016;7:91. 27014270 10.3389/fimmu.2016.00091 PMC4789404

[33] Zhang C , Oberoi P, Oelsner S, Waldmann A, Lindner A, Tonn T, . Chimeric antigen receptor-engineered NK-92 cells: an off-the-shelf cellular therapeutic for targeted elimination of cancer cells and induction of protective antitumor immunity. Front Immunol 2017;8:533. 28572802 10.3389/fimmu.2017.00533 PMC5435757

[34] Urlaub D , Hofer K, Muller ML, Watzl C. LFA-1 activation in NK cells and their subsets: influence of receptors, maturation, and cytokine stimulation. J Immunol 2017;198:1944–51. 28100681 10.4049/jimmunol.1601004

[35] Fauriat C , Long EO, Ljunggren HG, Bryceson YT. Regulation of human NK-cell cytokine and chemokine production by target cell recognition. Blood 2010;115:2167–76. 19965656 10.1182/blood-2009-08-238469 PMC2844017

[36] Robertson MJ . Role of chemokines in the biology of natural killer cells. J Leuk Biol 2002;71:173–83. 11818437

[37] Minas TZ , Surdez D, Javaheri T, Tanaka M, Howarth M, Kang H-J, . Combined experience of six independent laboratories attempting to create an Ewing sarcoma mouse model. Oncotarget 2017;8:34141–63. 27191748 10.18632/oncotarget.9388 PMC5470957

[38] Cillo AR , Mukherjee E, Bailey NG, Onkar S, Daley J, Salgado C, . Ewing sarcoma and osteosarcoma have distinct immune signatures and intercellular communication networks. Clin Cancer Res 2022;28:4968–82. 36074145 10.1158/1078-0432.CCR-22-1471 PMC9669190

[39] Verhoeven DHJ , de Hooge ASK, Mooiman ECK, Santos SJ, ten Dam MM, Gelderblom H, . NK cells recognize and lyse Ewing sarcoma cells through NKG2D and DNAM-1 receptor dependent pathways. Mol Immunol 2008;45:3917–25. 18657862 10.1016/j.molimm.2008.06.016

[40] Narasimhan PB , Eggert T, Zhu YP, Marcovecchio P, Meyer MA, Wu R, . Patrolling monocytes control NK cell expression of activating and stimulatory receptors to curtail lung metastases. J Immunol 2020;204:192–8. 31767784 10.4049/jimmunol.1900998 PMC7890694

[41] Cassetta L , Pollard JW. Cancer immunosurveillance: role of patrolling monocytes. Cell Res 2016;26:3–4. 26634605 10.1038/cr.2015.144 PMC4816132

[42] Sun R , Xiong Y, Liu H, Gao C, Su L, Weng J, . Tumor-associated neutrophils suppress antitumor immunity of NK cells through the PD-L1/PD-1 axis. Transl Oncol 2020;13:100825. 32698059 10.1016/j.tranon.2020.100825 PMC7372151

[43] Li P , Lu M, Shi J, Hua L, Gong Z, Li Q, . Dual roles of neutrophils in metastatic colonization are governed by the host NK cell status. Nat Commun 2020;11:4387. 32873795 10.1038/s41467-020-18125-0 PMC7463263

[44] Berghuis D , Santos SJ, Baelde HJ, Taminiau AH, Egeler RM, Schilham MW, . Pro-inflammatory chemokine-chemokine receptor interactions within the Ewing sarcoma microenvironment determine CD8(+) T-lymphocyte infiltration and affect tumour progression. J Pathol 2011;223:347–57. 21171080 10.1002/path.2819

[45] Woo SR , Corrales L, Gajewski TF. Innate immune recognition of cancer. Annu Rev Immunol 2015;33:445–74. 25622193 10.1146/annurev-immunol-032414-112043

[46] Galon J , Bruni D. Approaches to treat immune hot, altered and cold tumours with combination immunotherapies. Nat Rev Drug Discov 2019;18:197–218. 30610226 10.1038/s41573-018-0007-y

