# Supplementary material for: Natural Killer Cell Activation by Ubiquitin-specific Protease 6 Mediates Tumor Suppression in Ewing Sarcoma
Source: Cancer Res Commun. 2023 Aug 22;3(8):1615–27. doi: 10.1158/2767-9764.CRC-22-0505 (PMC10443598; doi:10.1158/2767-9764.CRC-22-0505)
Supplement: Supplementary Table S1 — Flow antibodies used [file crc-22-0505-s01.pdf]

Flow cytometry antibodies for human antigens

	Antigen	Fluorophore	Vendor	Cat No.	Clone	Dilution
1	CD45	APC	Biolegend	304012	HI30	1:20
2	CD69	BV605	Biolegend	310938	FN50	1:20
3	CD107a	BV510	Biolegend	328632	H4A3	1:20
4	CD54	BV510	BD	740170	HA58	1:100
5	CXCL9	APC	Biolegend	357905	J1015E10	1:20
6	CXCL10	PE	Biolegend	519503	J034D6	1:20
7	IFNy	PeCy7	Biolegend	502520	4S.B3	1:100
8	HA	Pe-Dazzle	Biolegend	901529	16B12	1:50
9	TRAIL	APC	Biolegend	308210	RIK-2	1:20
10	IFNyR	PE	Biolegend	308704	GIR-94	1:20
11	IFNyR	APC	Sino	10338-MM05-A	HG08OC1307	1:20
12	IFNabR	PerCP	R&D Systems	FAB245C	85228	1:10
13	DR5	APC	Biolegend	307408	DJR2-4	1:25
14	DR5	PE	Biolegend	307406	DJR2-4	1:20
15	MICAB	BV650	BD	742325	6D4	1:100
16	CD112	PeCy7	Biolegend	337414	TX31	1:20
17	CD155	BV605	BD	745215	TX24	1:50
18	ULBP256	AF700	Biolegend	165903	FAB1298N	1:50

Flow cytometry antibodies for mouse antigens

	Antigen	Fluorophore	Vendor	Cat No.	Clone	Dilution
1	CD45	AF700	Biolegend	103128	30-F11	1:100
2	CD45	APC-Cy7	Biolegend	103116	30-F11	1:100
3	NK1.1	BV605	Biolegend	108740	PK136	1:20
4	NK1.1	PeDaz	Biolegend	108748	PK136	1:100
5	NKp46	PE	Biolegend	137604	29A1.4	1:20
6	NKp46	BV786	Biolegend	137637	29A1.4	1:50
7	CD69	APC	Biolegend	104514	H1.2F3	1:20
8	CD107a	PeCy7	Biolegend	121620	1D4B	1:20
9	CD107a	APC-Cy7	Biolegend	121616	1D4B	1:20
10	CD107a	Pe-Dazzle	Biolegend	121624	1D4B	1:20
11	CD25	BV605	Biolegend	102036	PC61	1:100
12	CD25	PeDaz	Biolegend	102048	PC61	1:50
13	CD11b	PE	Biolegend	101208	M1/70	1:100
14	CD27	BV510	Biolegend	124229	LG.3A10	1:100
15	CD86	BV650	Biolegend	105036	GL-1	1:100
16	CD86	BV785	Biolegend	105043	GL-1	1:100
17	MHC-II	PB	Biolegend	107620	M5/114.15.2	1:100
18	TRAIL	PeCy7	Biolegend	109312	N2B2	1:20
19	Ly6C	BV510	Biolegend	128033	HK1.4	1:50
20	Ly6C	BV650	Biolegend	128049	HK1.4	1:50
21	Ly6G	AF700	Biolegend	127622	1A8	1:200
22	NKG2D	PE	Biolegend	115705	C7	1:100
23	CD11c	PerCp	Biolegend	117326	N418	1:100
24	F4/80	BV785	Biolegend	123141	BM8	1:50

Supplementary Table S1: Flow cytometry antibodies used in this study
