# Supplementary material for: Natural Killer Cell Activation by Ubiquitin-specific Protease 6 Mediates Tumor Suppression in Ewing Sarcoma
Source: Cancer Res Commun. 2023 Aug 22;3(8):1615–27. doi: 10.1158/2767-9764.CRC-22-0505 (PMC10443598; doi:10.1158/2767-9764.CRC-22-0505)
Supplement: Supplementary Figure S1 — NK depletion confirmation [file crc-22-0505-s02.pdf]

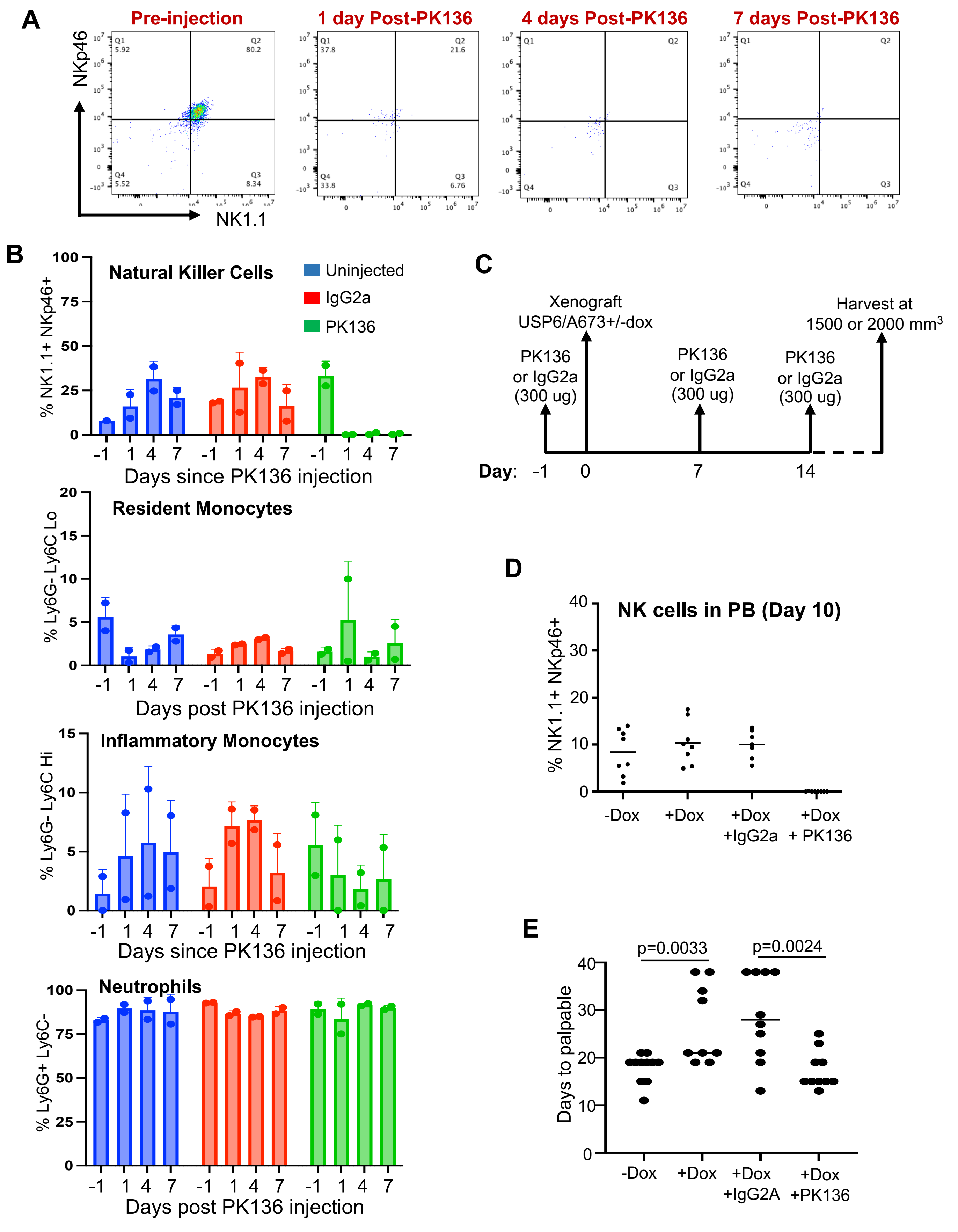

Supplementary Figure S1: NK depletion from RAG2-/- mice. A/B) To determine kinetics of NK depletion, RAG2-/- mice were injected intraperitoneally with 300ug of anti-NK1.1 (PK136). Peripheral blood (PB) was collected 1 day prior to injection, and 1, 4, and 7 days post-injection, and NK levels quantified by surface NK1.1 and NKp46 (pilot study n=2 mice). **B**) Levels of indicated lineages were quantified from PB using the indicated markers. **C**) Experimental design to assess effects of NK cell depletion on tumor growth (n=10 mice/cohort). **D**) NK cell levels were assessed on Days 10 (n=8/cohort). **E**) Time required for tumors to become palpable (approximately 200-300 mm^3) was determined.
