# Supplementary material for: Natural Killer Cell Activation by Ubiquitin-specific Protease 6 Mediates Tumor Suppression in Ewing Sarcoma
Source: Cancer Res Commun. 2023 Aug 22;3(8):1615–27. doi: 10.1158/2767-9764.CRC-22-0505 (PMC10443598; doi:10.1158/2767-9764.CRC-22-0505)
Supplement: Supplementary Figure S2 — Gating for NK populations and intratumoral quantification [file crc-22-0505-s03.pdf]

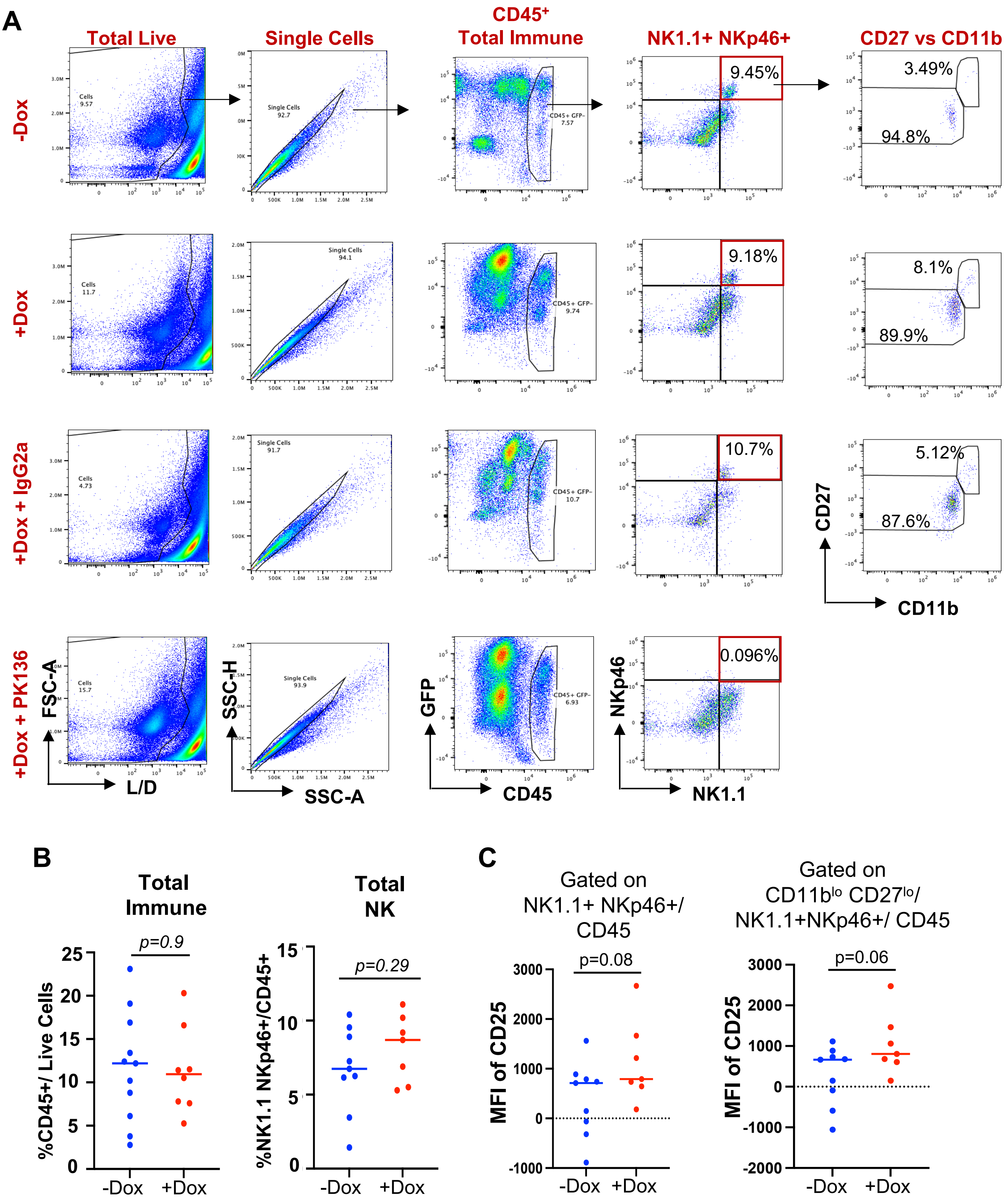

Supplementary Figure S2: USP6 effects on total immune and NK cell infiltration **A)** Gating strategy for total NK cells (NK1.1+ NKp46+), and NK subpopulations (defined by CD27 and CD11b expression). **B/C)** Flow cytometry was performed on digested tumors to quantify: **B**) Abundance of total immune and NK cells, and **C)** Proliferative potential of NK cells (as monitored by CD25, the IL2 Receptor α) (-Dox n=11, +Dox n=8).
