# Supplementary material for: Natural Killer Cell Activation by Ubiquitin-specific Protease 6 Mediates Tumor Suppression in Ewing Sarcoma
Source: Cancer Res Commun. 2023 Aug 22;3(8):1615–27. doi: 10.1158/2767-9764.CRC-22-0505 (PMC10443598; doi:10.1158/2767-9764.CRC-22-0505)
Supplement: Supplementary Figure S3 — Primary mouse and human NK killing assays [file crc-22-0505-s04.pdf]

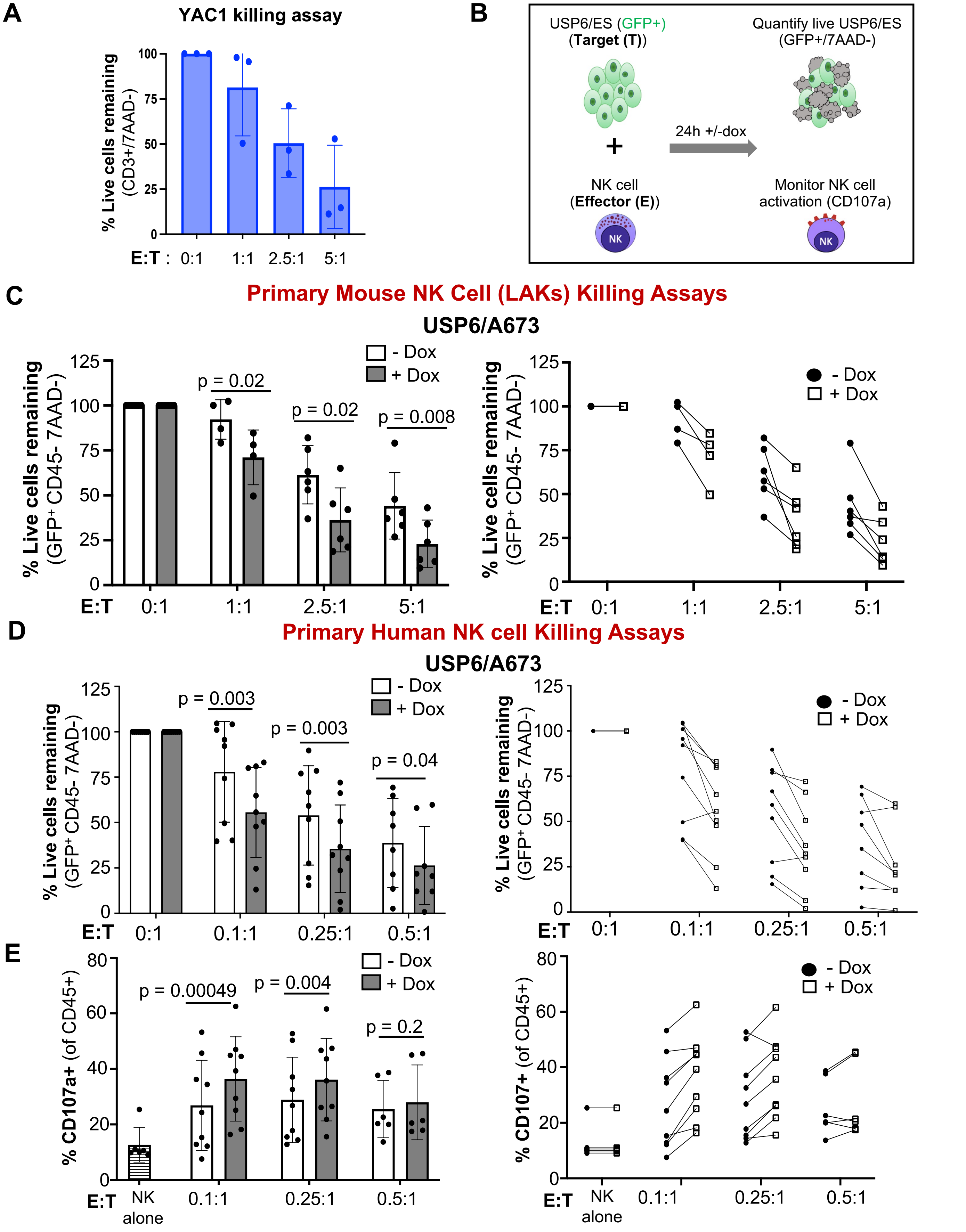

Supplementary Figure S3: USP6 enhances cytolytic activity and activation of primary mouse NK (LAKs) and primary human NK cells **A)** Spleen-derived LAKs were co-cultured with mouse YAC1 cells at the indicated E:T ratios for 24h, and percent live remaining YAC1 cells was quantified to confirm LAK cytolytic activity (n=3). **B)** Assay for monitoring cytolytic activity and NK cell activation. **C)** Cytolytic activity of LAKs against USP6/A673 was determined. Right, each dot pair represents a distinct LAKs preparation (each comprising spleens from 3 mice), incubated with target cells with or without dox (n=4-6). **D/E**) Primary human NK cells and USP6/A673 were co-cultured with or without dox. Cytolytic activity was measured in **D**, and degranulation (surface CD107a) in **E**. Data are from 5 independent human NK donors, with samples assayed in singlicate or duplicate depending on cell availability.
