# Supplementary material for: Natural Killer Cell Activation by Ubiquitin-specific Protease 6 Mediates Tumor Suppression in Ewing Sarcoma
Source: Cancer Res Commun. 2023 Aug 22;3(8):1615–27. doi: 10.1158/2767-9764.CRC-22-0505 (PMC10443598; doi:10.1158/2767-9764.CRC-22-0505)
Supplement: Supplementary Figure S4 — RT-qPCR of surface markers induced by USP6 [file crc-22-0505-s05.pdf]

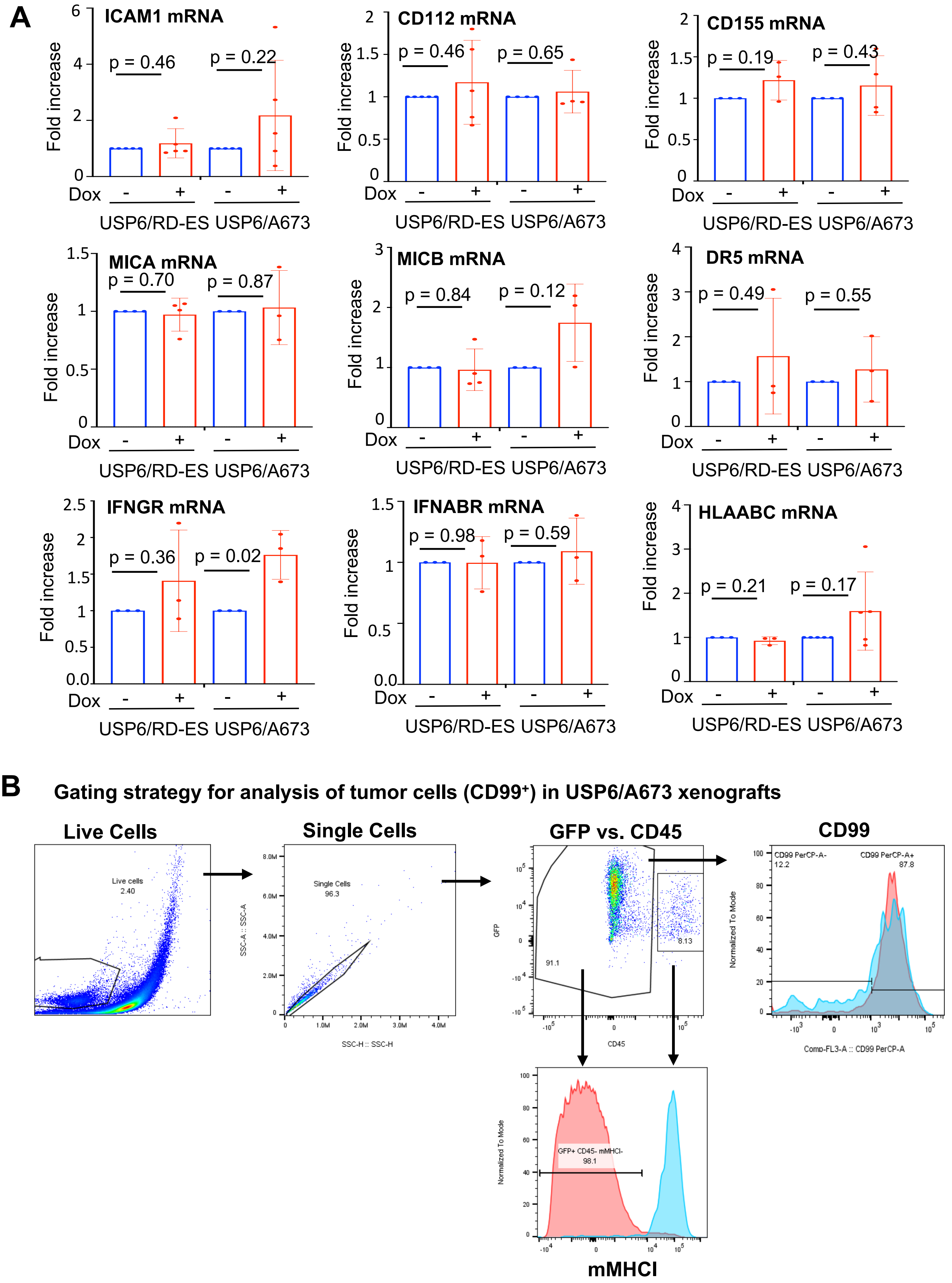

Supplementary Figure S4: Effects of USP6 on mRNA levels of NK-activating ligands and surface markers A) USP6/A673 or USP6/RD-ES cells were treated with or without dox, and RT-qPCR was performed for the indicated genes (n=3-5). **B**) Gating strategy for tumor cells in USP6/A673 xenografts. Tumor cells are identified as human CD99-positive and mouse MHCI-negative.
