# Supplementary material for: Natural Killer Cell Activation by Ubiquitin-specific Protease 6 Mediates Tumor Suppression in Ewing Sarcoma
Source: Cancer Res Commun. 2023 Aug 22;3(8):1615–27. doi: 10.1158/2767-9764.CRC-22-0505 (PMC10443598; doi:10.1158/2767-9764.CRC-22-0505)
Supplement: Supplementary Figure S5 — Paracrine feedforward loop between NK and USP6-A673 cells [file crc-22-0505-s06.pdf]

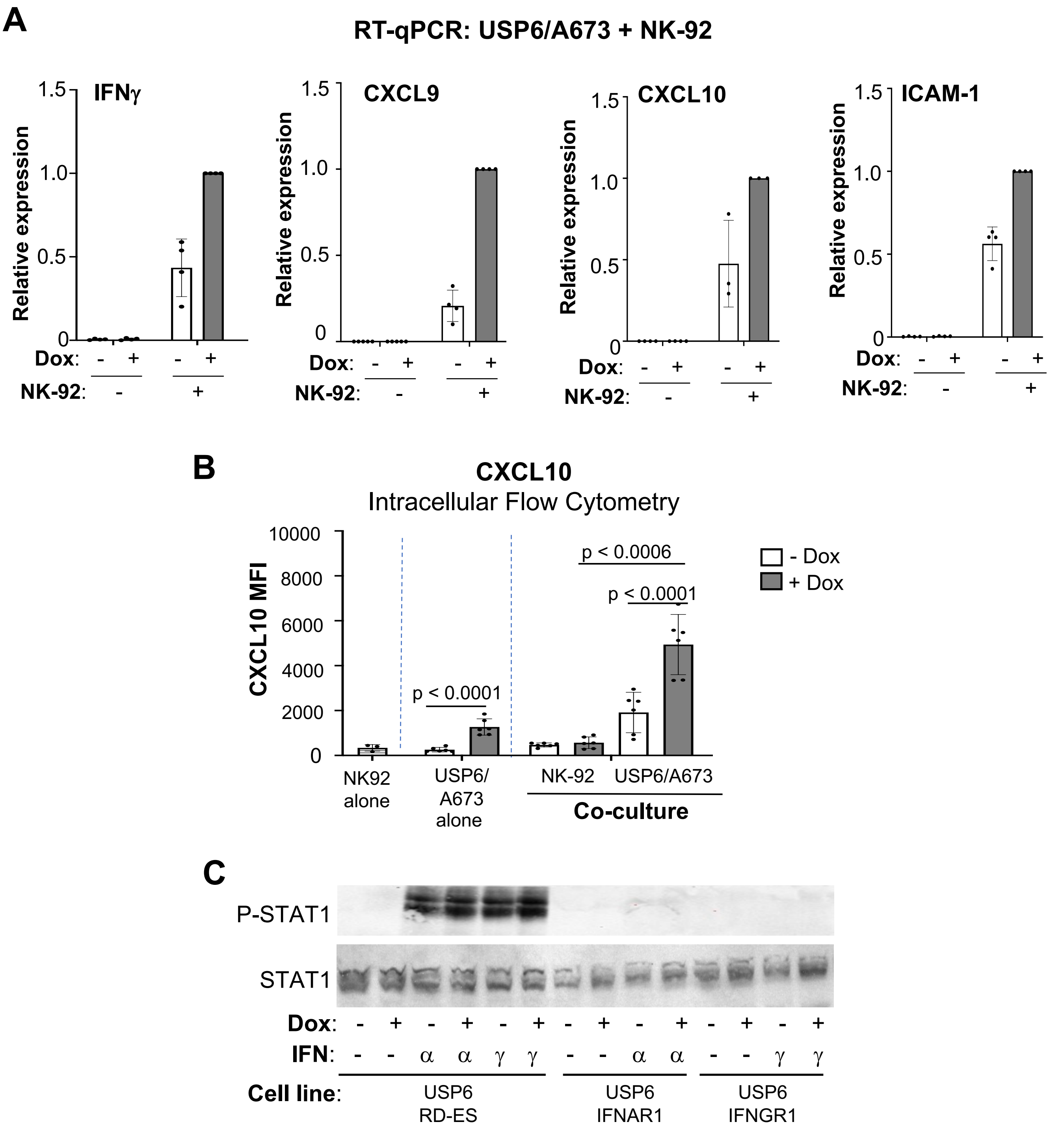

Supplementary Figure S5: Paracrine feedforward loop is mediated through IFNGR signaling in ES cells NK-92 and USP6/A673 were co-cultured at 0.1:1 ratio in the absence or presence of dox. **A)** RT-qPCR was performed for the indicated genes (n=3). **B**) Intracellular flow cytometry was performed to quantify CXCL10 protein levels (n=3). **C**) Functional inactivation of the IFNAR1 and IFNGR1 in CRISPR RD-ES cells was confirmed by treating cells with IFNα or IFNγ (5ng/ml) for 30 min, then probing lysates for total STAT1 and phospho-STAT1 by western.
