# Supplementary material for: Natural Killer Cell Activation by Ubiquitin-specific Protease 6 Mediates Tumor Suppression in Ewing Sarcoma
Source: Cancer Res Commun. 2023 Aug 22;3(8):1615–27. doi: 10.1158/2767-9764.CRC-22-0505 (PMC10443598; doi:10.1158/2767-9764.CRC-22-0505)
Supplement: Supplementary Figure S6 — Gating strategy for NK cells in peripheral blood [file crc-22-0505-s07.pdf]

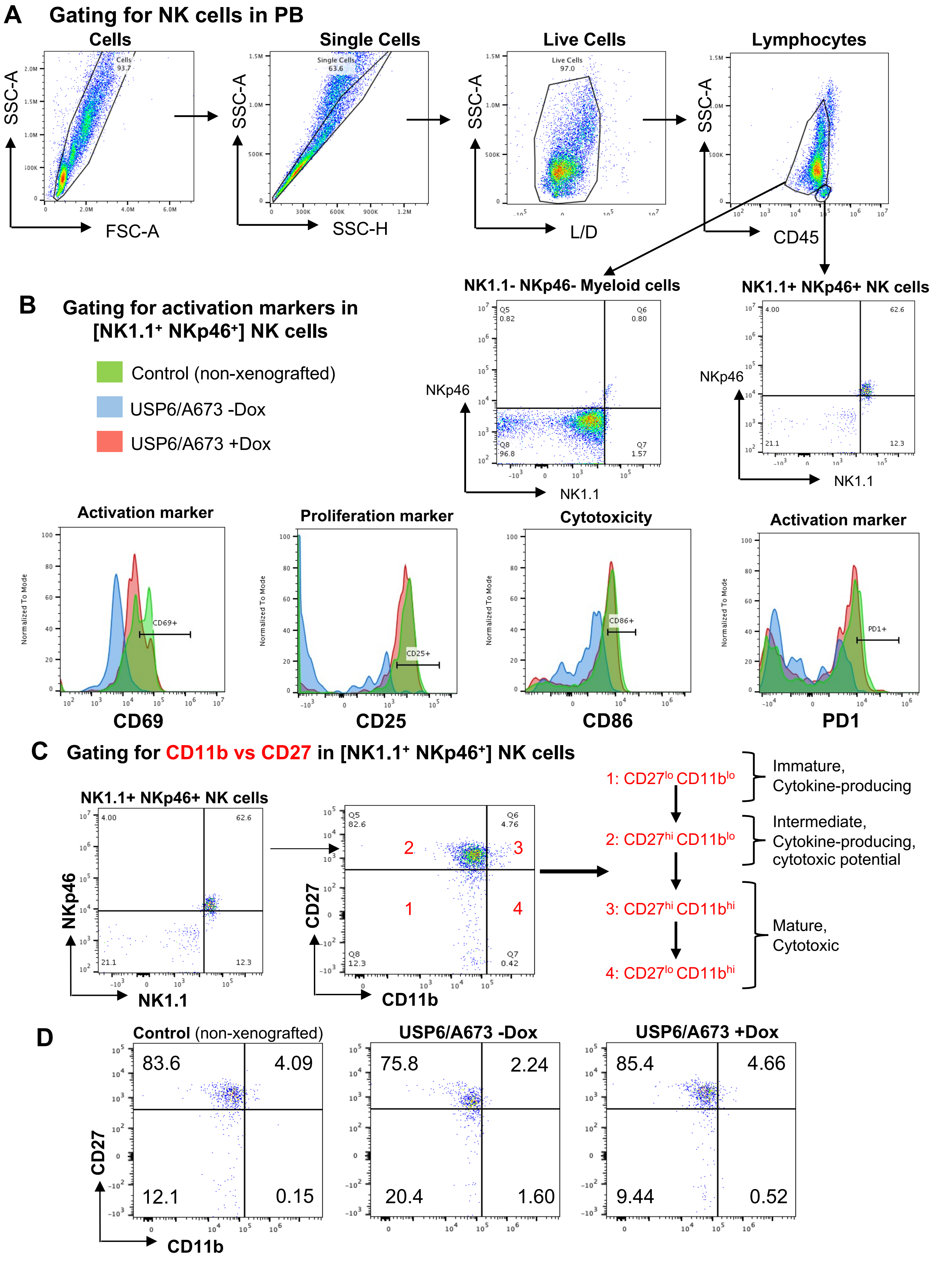

Supplementary Figure S6: Gating strategy for monitoring NK populations in peripheral blood **A)** Gating strategy for NK cells (NK1.1+ NKp46+) in PB. **B)** Histograms for the indicated markers of NK activation and function. **C)** Gating strategy for CD27 vs. CD11b in NK cells. Right, functional characteristics of the four populations. **D**) CD27/CD11b staining in NKs from PB of Control RAG2-/- mice vs. xenografted with USP6/A673 in the absence or presence of dox treatment.
