# Supplementary material for: Natural Killer Cell Activation by Ubiquitin-specific Protease 6 Mediates Tumor Suppression in Ewing Sarcoma
Source: Cancer Res Commun. 2023 Aug 22;3(8):1615–27. doi: 10.1158/2767-9764.CRC-22-0505 (PMC10443598; doi:10.1158/2767-9764.CRC-22-0505)
Supplement: Supplementary Figure S7 — Gating strategy for myeloid lineages in peripheral blood [file crc-22-0505-s08.pdf]

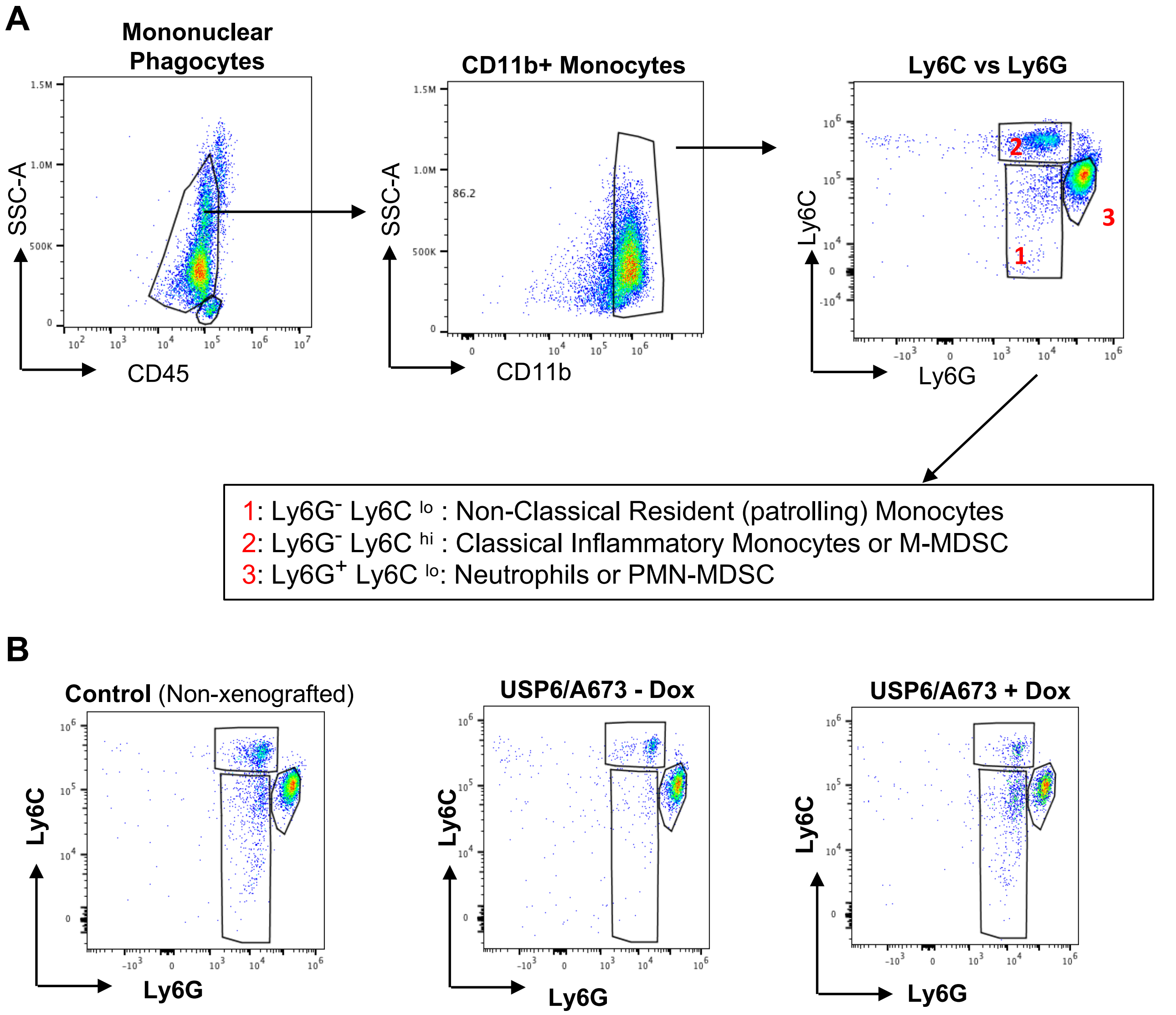

Supplementary Figure S7: Gating strategy for myeloid lineages in peripheral blood A) Ly6C and Ly6G levels were assessed in the CD11b+/CD45+ population; populations 1-3 are defined. **B**) Ly6C/Ly6G staining in CD11b+/CD45+ cells from PB of Control RAG2-/- mice vs. xenografted with USP6/A673 in the absence or presence of dox treatment.
