# Supplementary material for: Natural Killer Cell Activation by Ubiquitin-specific Protease 6 Mediates Tumor Suppression in Ewing Sarcoma
Source: Cancer Res Commun. 2023 Aug 22;3(8):1615–27. doi: 10.1158/2767-9764.CRC-22-0505 (PMC10443598; doi:10.1158/2767-9764.CRC-22-0505)
Supplement: Supplementary Figure S8 — Gating strategy for immune lineages in abscopal response experiment [file crc-22-0505-s09.pdf]

Gating for NK cells in Distal Tumors

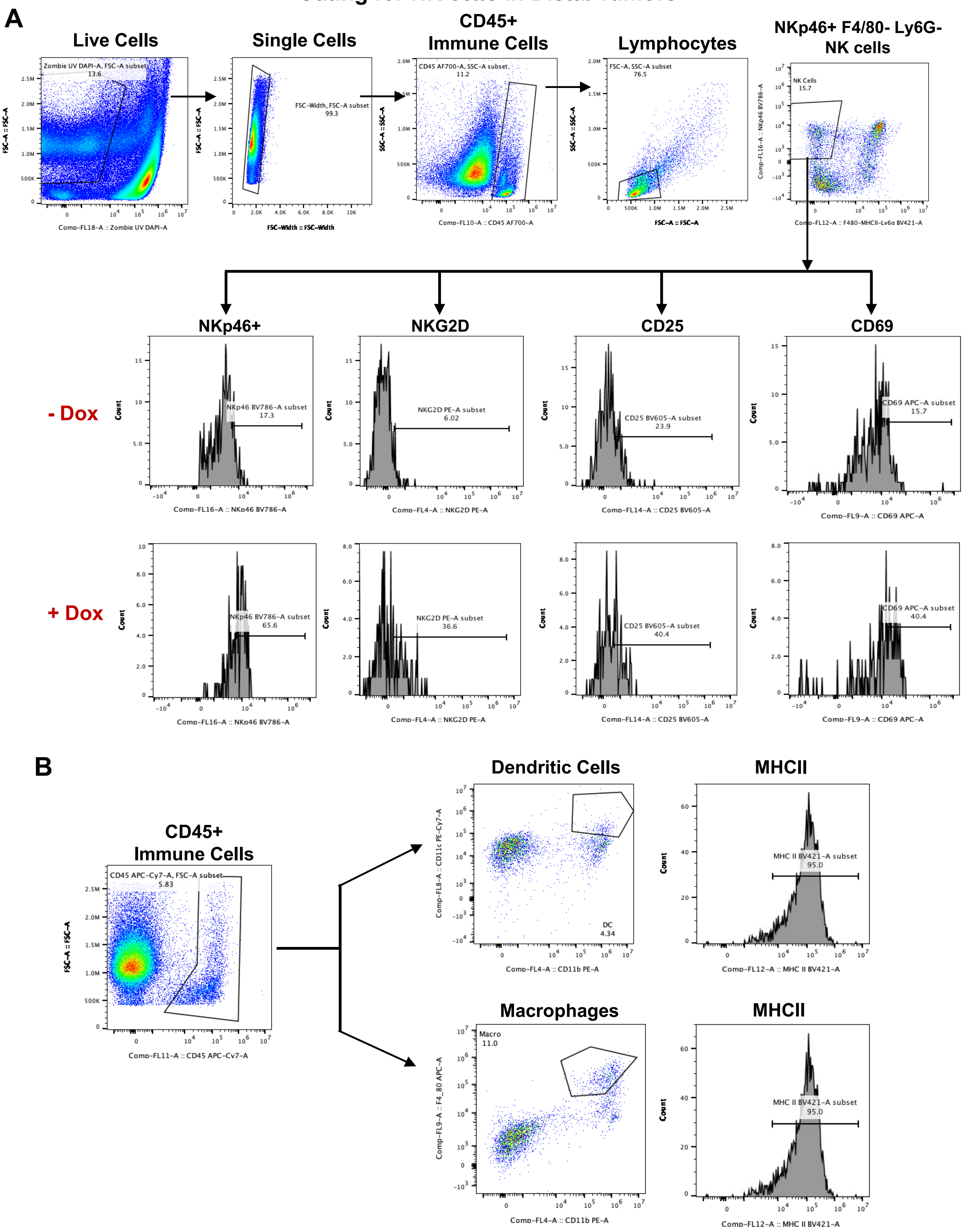

Supplementary Figure S8: **Gating strategy for NK cells in distal tumors**. **A**) Gating strategy for NK cells in distal tumors, and surface levels of the indicated markers. **B**) Gating strategy for dendritic cells and macrophages, and MHCII in these populations.
